# Supplementary figures and images for: The C. difficile clnRAB operon initiates adaptations to the host environment in response to LL-37
Source: PLoS Pathog. 2018 Aug 20;14(8):e1007153. doi: 10.1371/journal.ppat.1007153 (PMC6117091; doi:10.1371/journal.ppat.1007153)

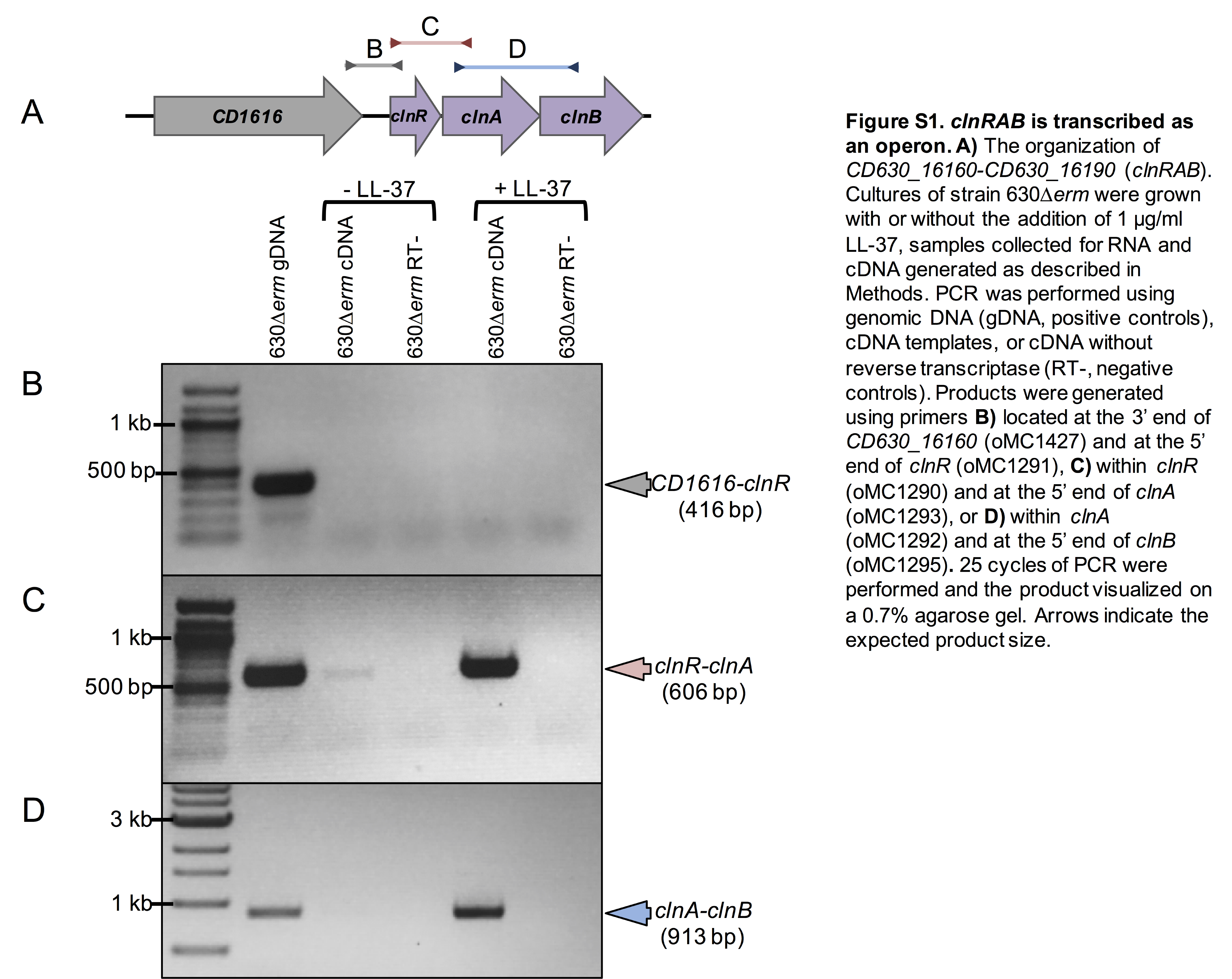

Supplement: S1 Fig — A) The organization of CD630_16160-CD630_16190 (clnRAB). Cultures of strain 630Δerm were grown with or without the addition of 1 μg/ml LL-37, samples collected for RNA and cDNA generated as described in Methods. PCR was performed using genomic DNA (gDNA, positive controls), cDNA templates, or cDNA without reverse transcriptase (RT-, negative controls). Products were generated using primers B) located at the 3’ end of CD630_16160 (oMC1427) and at the 5’ end of clnR (oMC1291), C) within clnR (oMC1290) and at the 5’ end of clnA (oMC1293), or D) within clnA (oMC1292) and at the 5’ end of clnB (oMC1295). 25 cycles of PCR were performed and the product visualized on a 0.7% agarose gel. Arrows indicate the expected product size. (TIF) [file ppat.1007153.s001.tif]

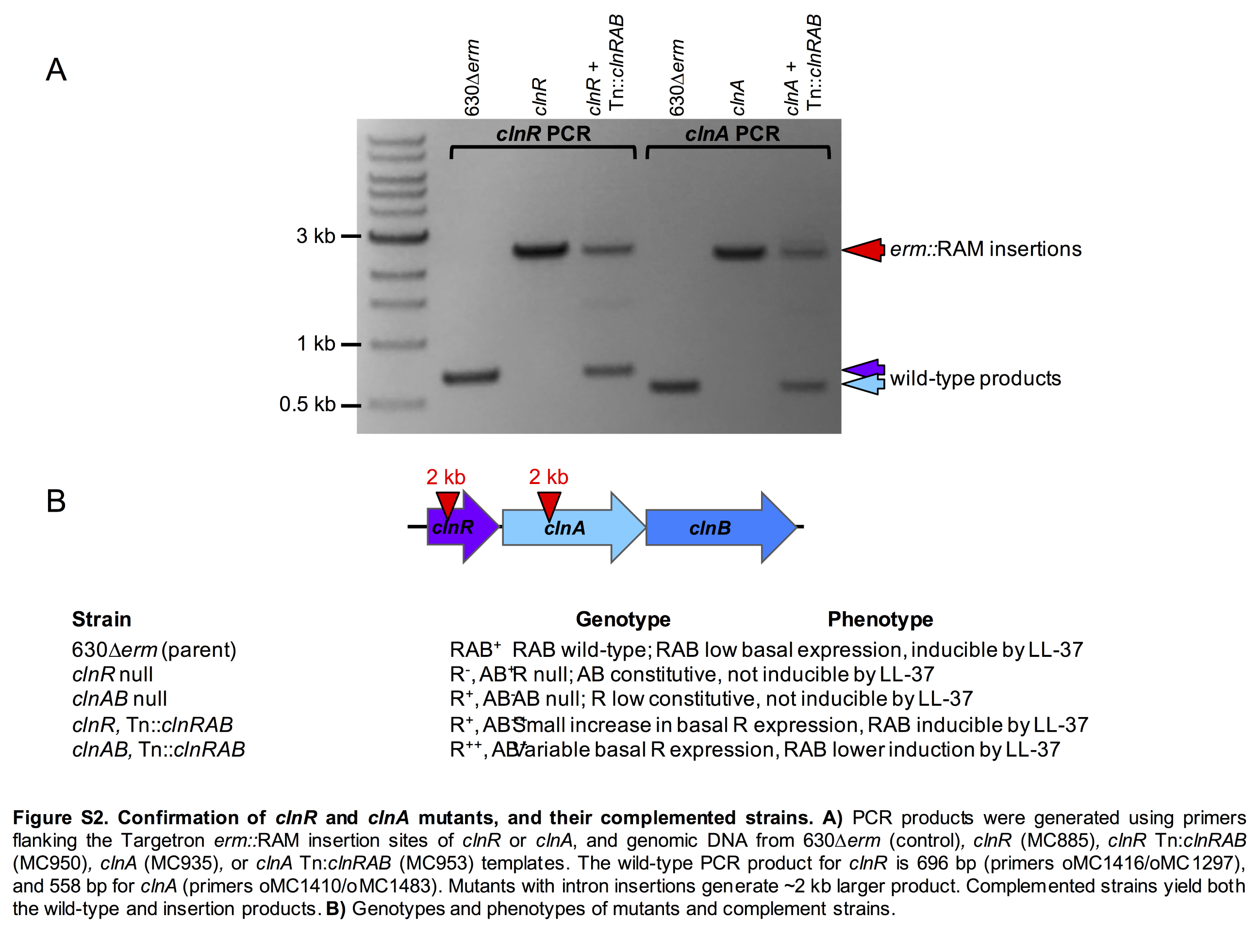

Supplement: S2 Fig — A) PCR products were generated using primers flanking the Targetron erm::RAM insertion sites of clnR or clnA, and genomic DNA from 630Δerm (control), clnR (MC885), clnR Tn:clnRAB (MC950), clnA (MC935), or clnA Tn:clnRAB (MC953) templates. The wild-type PCR product for clnR is 696 bp (primers oMC1416/oMC1297), and 558 bp for clnA (primers oMC1410/oMC1483). Mutants with intron insertions generate ~2 kb larger product. Complemented strains yield both the wild-type and insertion products. B) Genotypes and phenotypes of mutants and complement strains. (TIF) [file ppat.1007153.s002.tif]

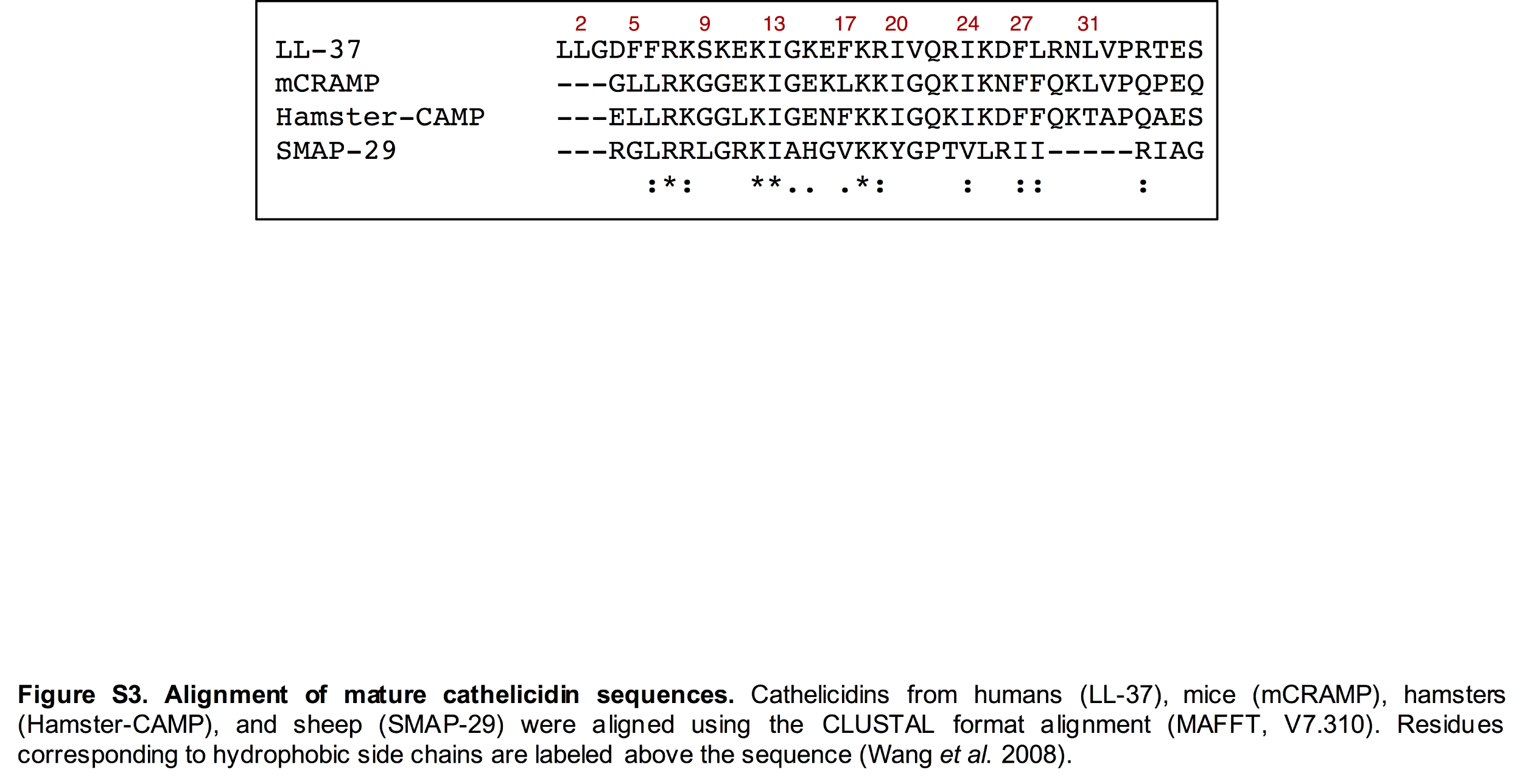

Supplement: S3 Fig — Cathelicidins from humans (LL-37), mice (mCRAMP) and sheep (SMAP-29) were aligned using the CLUSTAL format alignment (MAFFT, V7.310). Residues corresponding to hydrophobic side chains are labeled above the sequence (Wang et al. 2008). (TIF) [file ppat.1007153.s003.tif]

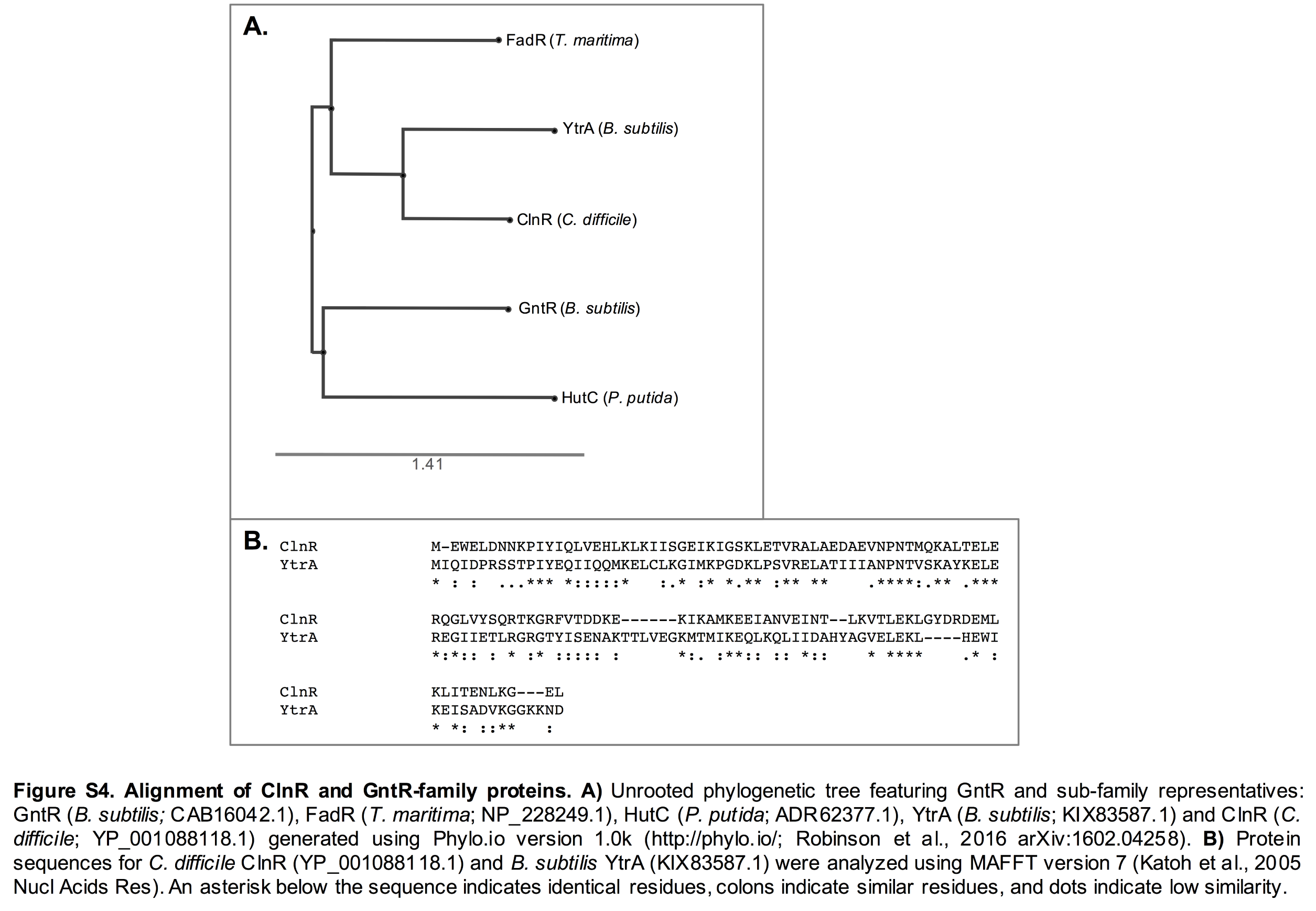

Supplement: S4 Fig — A) Unrooted phylogenetic tree featuring GntR and sub-family representatives: GntR (B. subtilis; CAB16042.1), FadR (T. maritima; NP_228249.1), HutC (P. putida; ADR62377.1), YtrA (B. subtilis; KIX83587.1) and ClnR (C. difficile; YP_001088118.1) generated using Phylo.io version 1.0k (http://phylo.io/; Robinson et al., 2016 arXiv:1602.04258). B) Protein sequences for C. difficile ClnR (YP_001088118.1) and B. subtilis YtrA (KIX83587.1) were analyzed using MAFFT version 7 (Katoh et al., 2005 Nucl Acids Res). An asterisk below the sequence indicates identical residues, colons indicate similar residues, and dots indicate low similarity. (TIF) [file ppat.1007153.s004.tif]

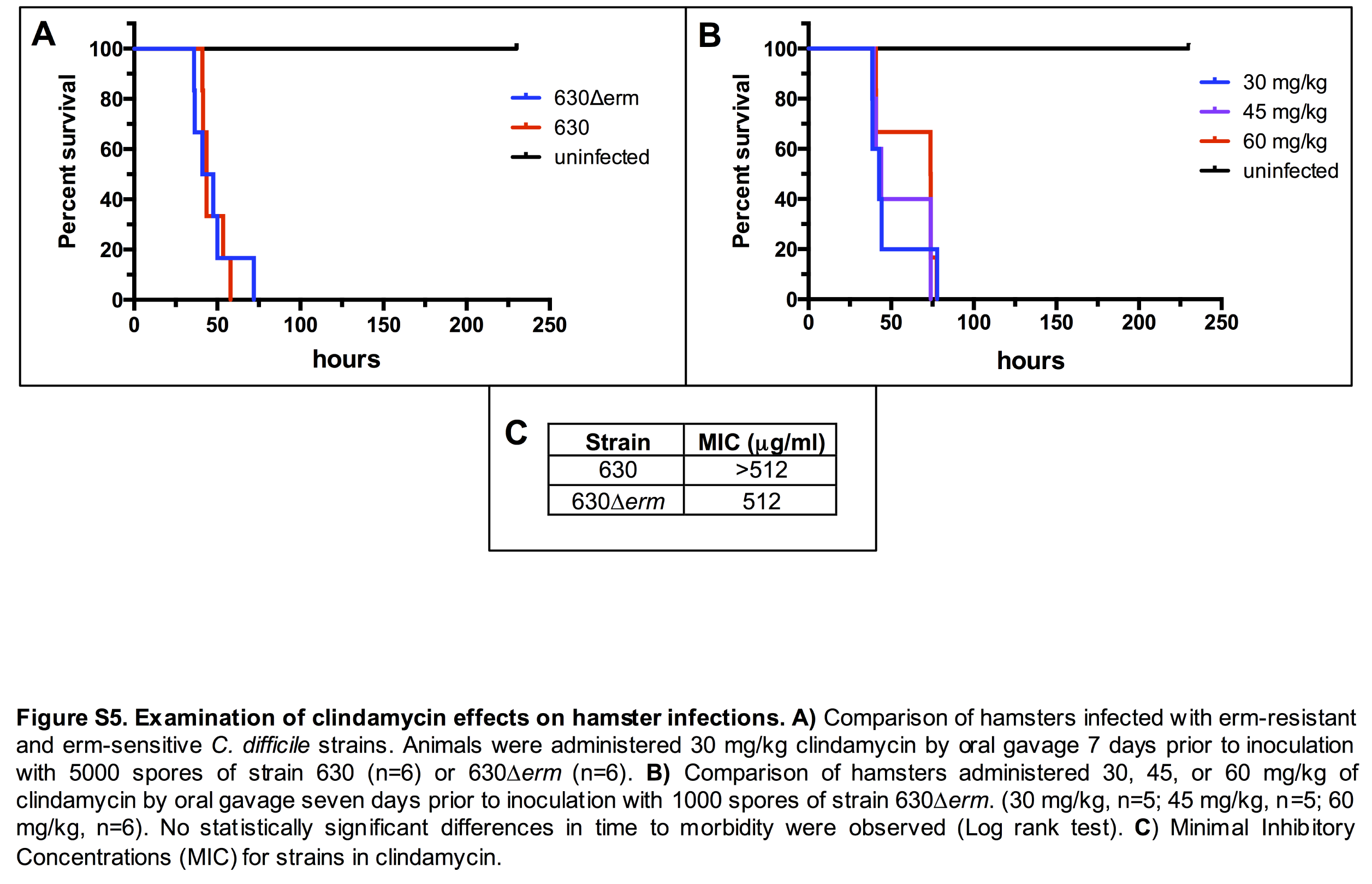

Supplement: S5 Fig — A) Comparison of hamsters infected with erm-resistant and erm-sensitive C. difficile strains. Animals were administered 30 mg/kg clindamycin by oral gavage 7 days prior to inoculation with 5000 spores of strain 630 (n = 6) or 630Δerm (n = 6). B) Comparison of hamsters administered 30, 45, or 60 mg/kg of clindamycin by oral gavage seven days prior to inoculation with 1000 spores of strain 630Δerm. (30 mg/kg, n = 5; 45 mg/kg, n = 5; 60 mg/kg, n = 6). No statistically significant differences in time to morbidity were observed (Log rank test). C) Minimal Inhibitory Concentrations (MIC) for strains in clindamycin. (TIF) [file ppat.1007153.s005.tif]

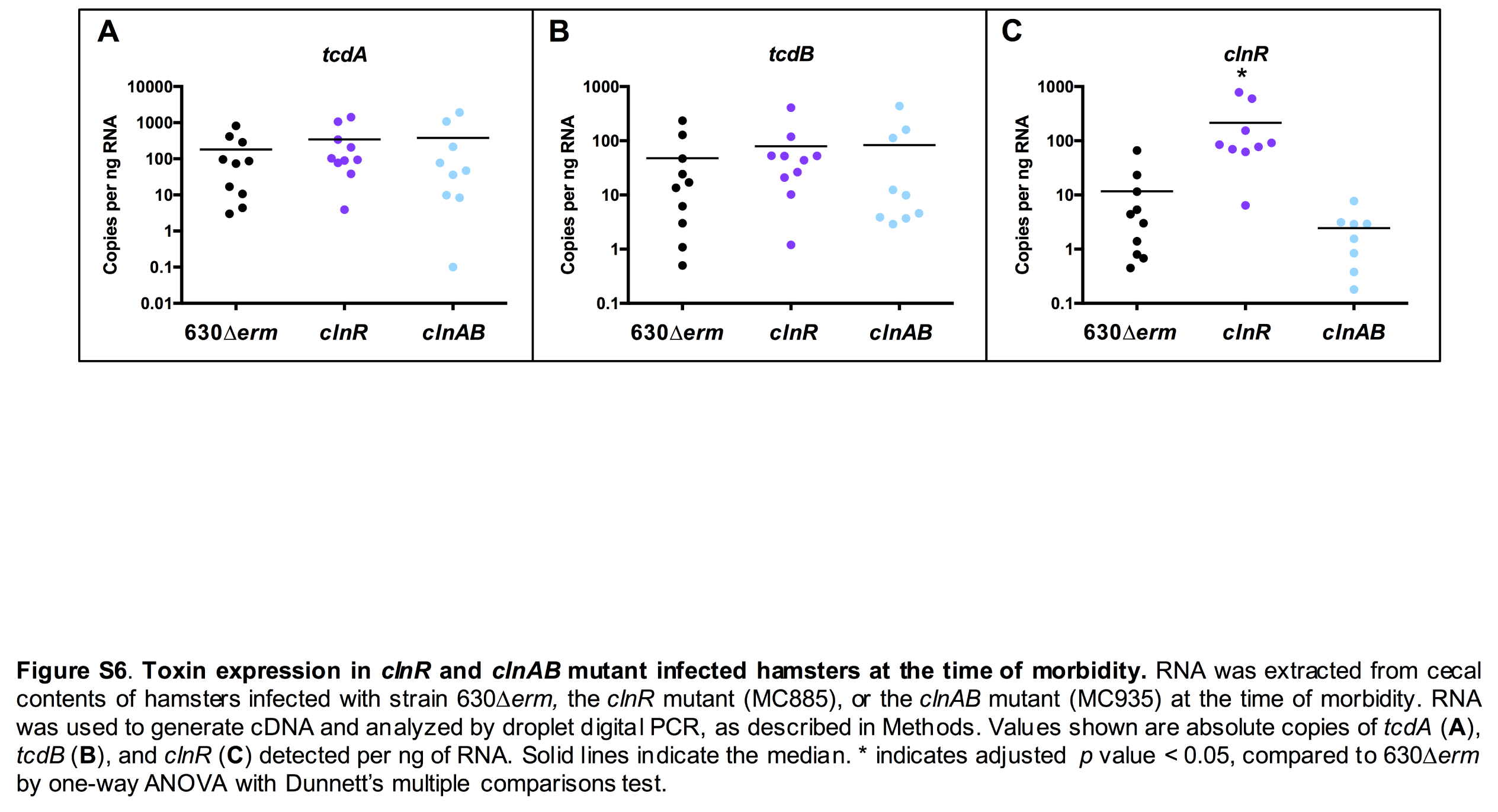

Supplement: S6 Fig — RNA was extracted from cecal contents of hamsters infected with strain 630Δerm, the clnR mutant (MC885), or the clnAB mutant (MC935) at the time of morbidity. RNA was used to generate cDNA and analyzed by droplet digital PCR, as described in Methods. Values shown are absolute copies of tcdA (A), tcdB (B), and clnR (C) detected per ng of RNA. Solid lines indicate the median. * indicates adjusted p value < 0.05, compared to 630Δerm by one-way ANOVA with Dunnett’s multiple comparisons test. (TIF) [file ppat.1007153.s006.tif]

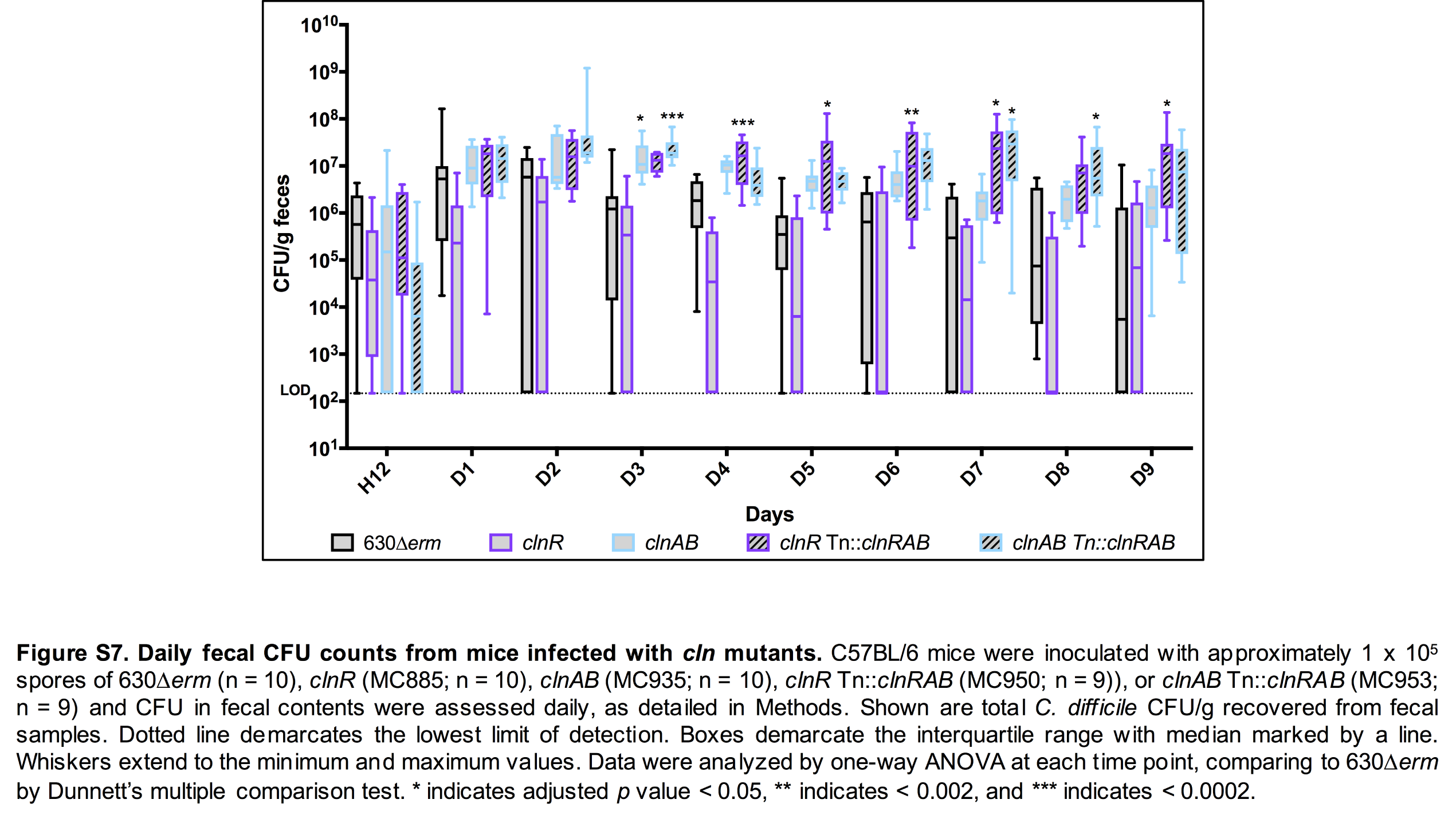

Supplement: S7 Fig — C57BL/6 mice were inoculated with approximately 1 x 10^5^ spores of 630Δerm (n = 10), clnR (MC885; n = 10), clnAB (MC935; n = 10), clnR Tn::clnRAB (MC950; n = 9)), or clnAB Tn::clnRAB (MC953; n = 9) and CFU in fecal contents were assessed daily, as detailed in Methods. Shown are total C. difficile CFU recovered from fecal samples. Dotted line demarcates the lowest limit of detection. Boxes demarcate the interquartile range with median marked by a line. Whiskers extend to the minimum and maximum values. Data were analyzed by one-way ANOVA at each time point, comparing to 630Δerm by Dunnett’s multiple comparison test. * indicates adjusted p value < 0.05, ** indicates < 0.002, and *** indicates < 0.0002. (TIF) [file ppat.1007153.s007.tif]

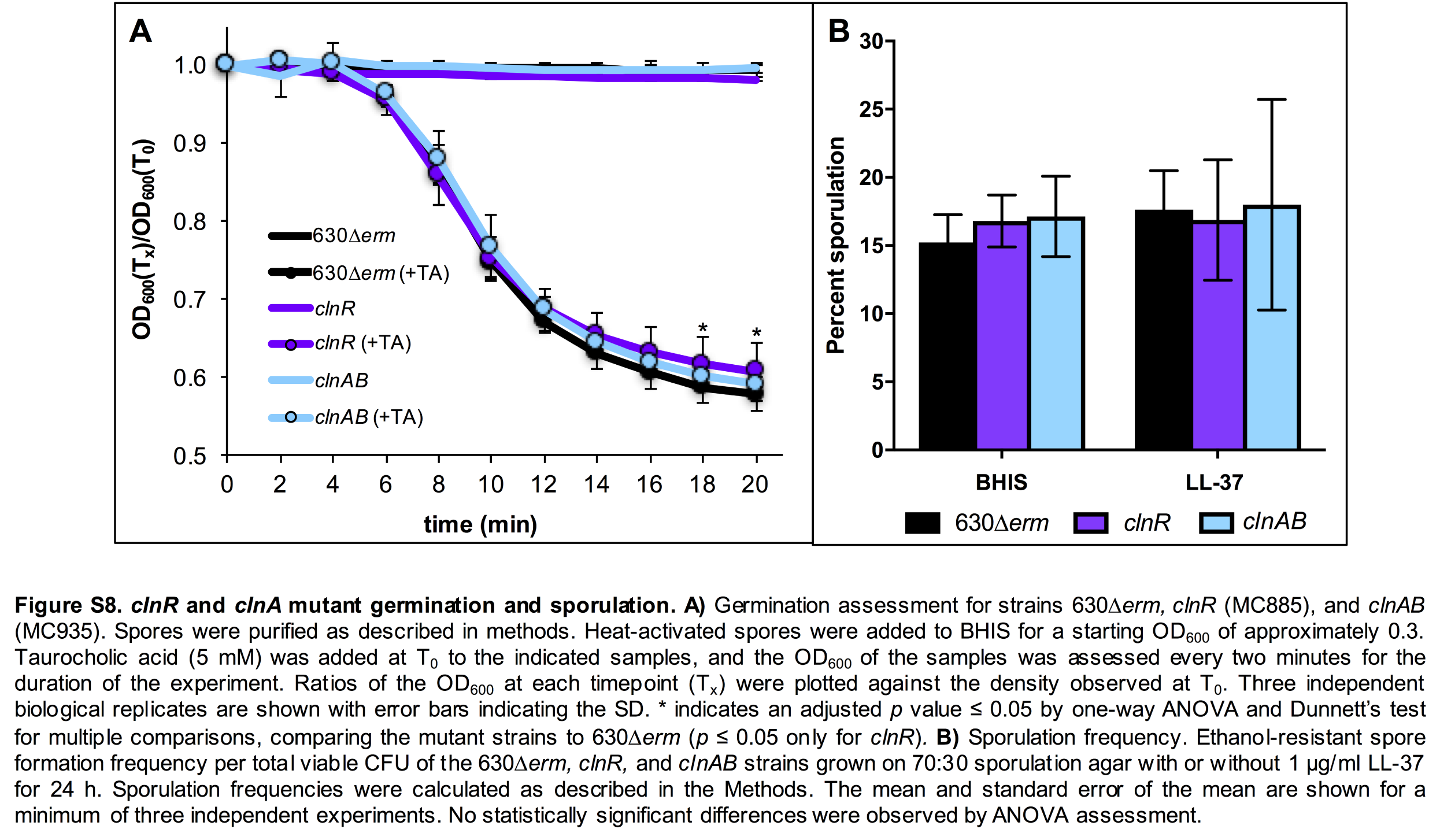

Supplement: S8 Fig — A) Germination assessment for strains 630Δerm, clnR (MC885), and clnAB (MC935). Spores were purified as described in methods. Heat-activated spores were added to BHIS for a starting OD600 of approximately 0.3. Taurocholic acid (5 mM) was added at T0 to the indicated samples, and the OD600 of the samples was assessed every two minutes for the duration of the experiment. Ratios of the OD600 at each timepoint (Tx) were plotted against the density observed at T0. Three independent biological replicates are shown with error bars indicating the SD. * indicates an adjusted p value ≤ 0.05 by one-way ANOVA and Dunnett’s test for multiple comparisons, comparing the mutant strains to 630Δerm (p ≤ 0.05 only for clnR). B) Sporulation frequency. Ethanol-resistant spore formation frequency per total viable CFU of the 630Δerm, clnR, and clnAB strains grown on 70:30 sporulation agar with or without 1 μg/ml LL-37 for 24 h. Sporulation frequencies were calculated as described in the Methods. The mean and standard error of the mean are shown for a minimum of three independent experiments. No statistically significant differences were observed by ANOVA assessment. (TIF) [file ppat.1007153.s008.tif]

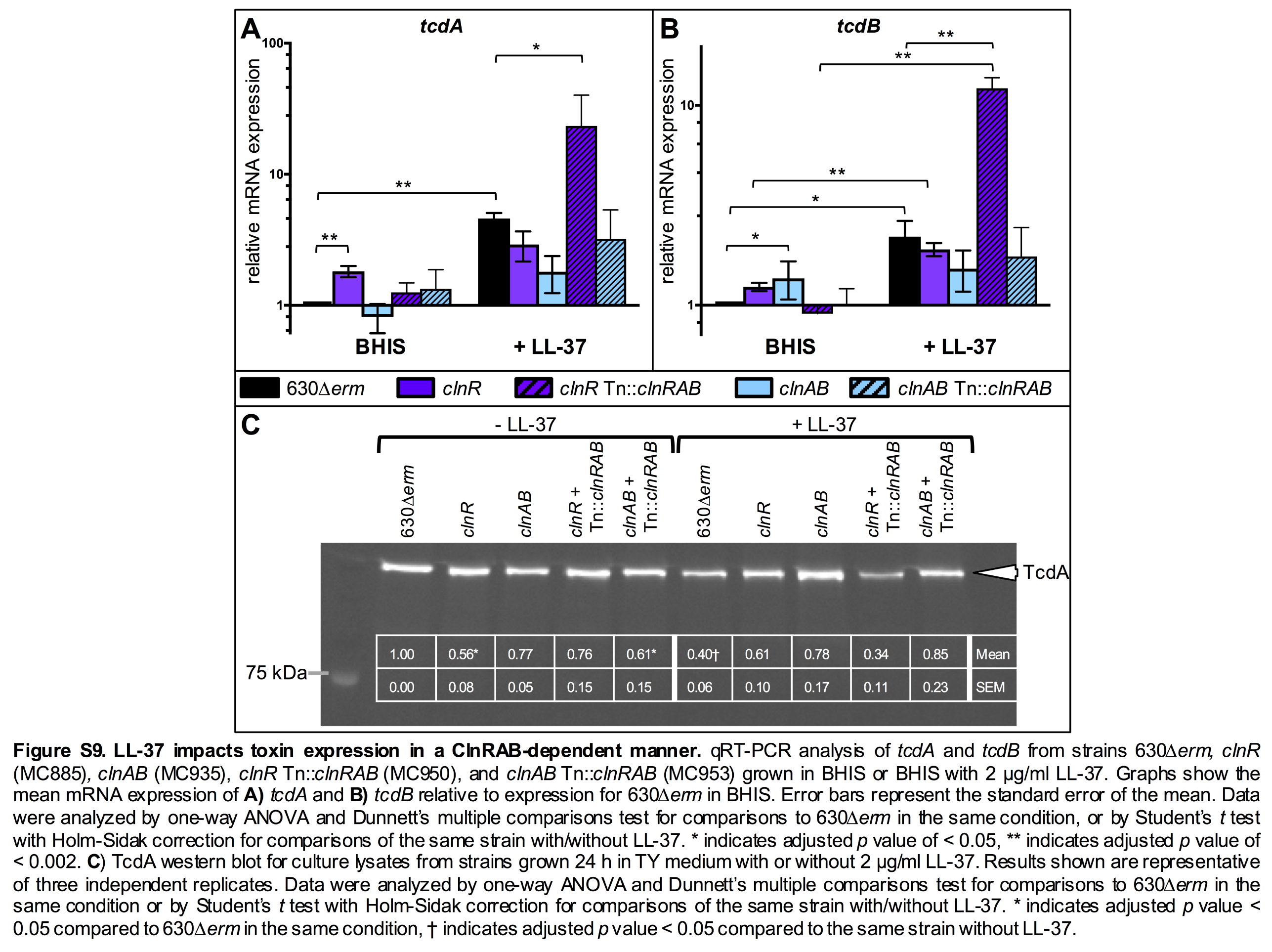

Supplement: S9 Fig — qRT-PCR analysis of tcdA and tcdB from strains 630Δerm, clnR (MC885), clnAB (MC935), clnR Tn::clnRAB (MC950), and clnAB Tn::clnRAB (MC953) grown in BHIS or BHIS with 2 μg/ml LL-37. Graphs show the mean mRNA expression of A) tcdA and B) tcdB relative to expression for 630Δerm in BHIS. Error bars represent the standard error of the mean. Data were analyzed by one-way ANOVA and Dunnett’s multiple comparisons test for comparisons to 630Δerm in the same condition, or by Student’s t-test with Holm-Sidak correction for comparisons of the same strain with/without LL-37. * indicates adjusted p value of < 0.05, ** indicates adjusted P value of < 0.002. C) TcdA western blot for culture lysates from strains grown 24 h in TY medium with or without 2 μg/ml LL-37. Results shown are representative of three independent replicates. Data were analyzed by one-way ANOVA and Dunnett’s multiple comparisons test for comparisons to 630Δerm in the same condition or by Student’s t test with Holm-Sidak correction for comparisons of the same strain with/without LL-37. * indicates adjusted p value < 0.05 compared to 630Δerm in the same condition, † indicates adjusted p value < 0.05 compared to the same strain without LL-37. (TIF) [file ppat.1007153.s009.tif]

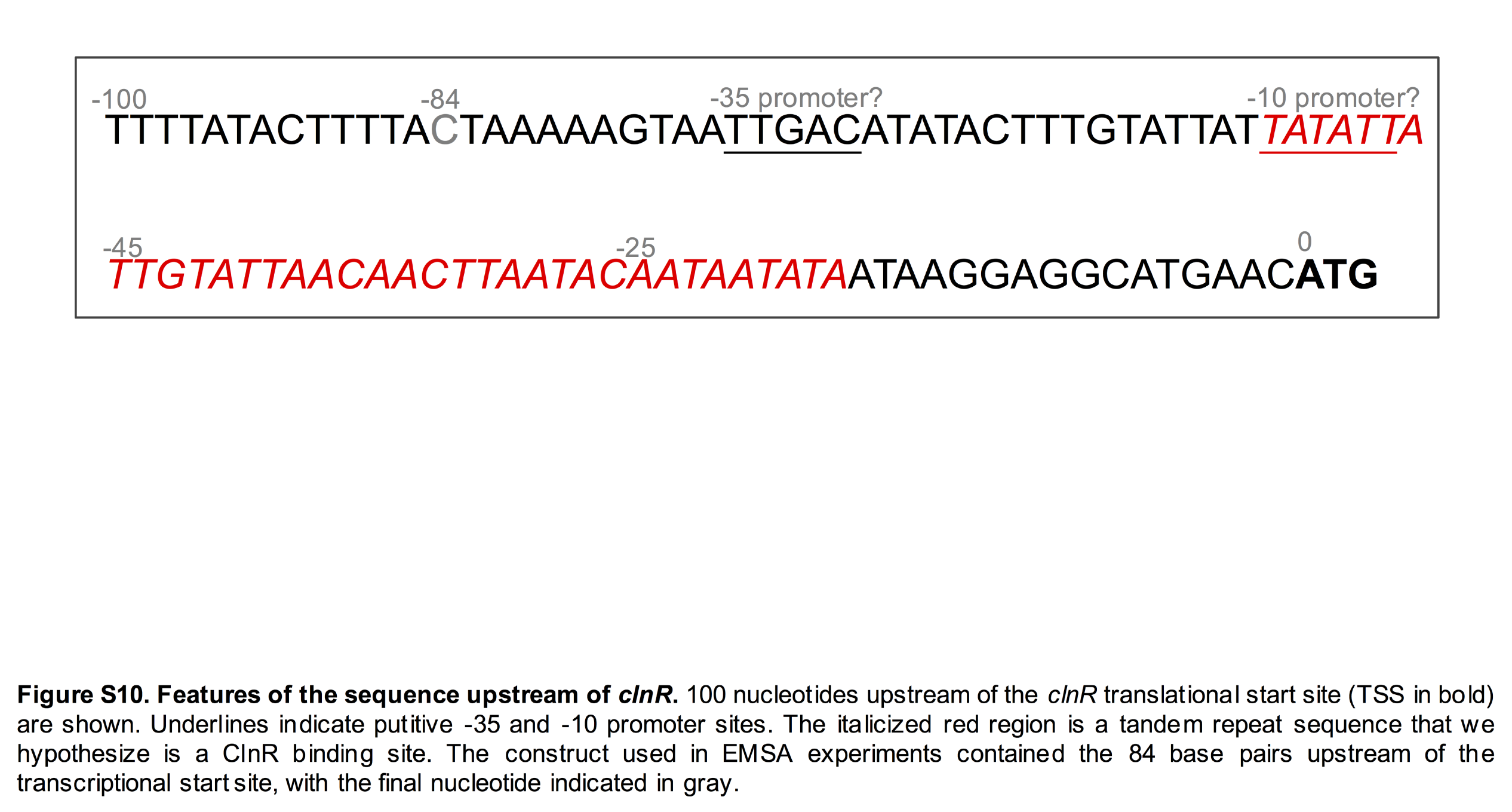

Supplement: S10 Fig — 100 nucleotides upstream of the clnR translational start site (TSS in bold) are shown. Underlines indicate putitive -35 and -10 promoter sites. The italicized red region is a tandem repeat sequence that we hypothesize is a ClnR binding site. The construct used in EMSA experiments contained the 84 base pairs upstream of the transcriptional start site, with the final nucleotide indicated in gray. (TIF) [file ppat.1007153.s010.tif]

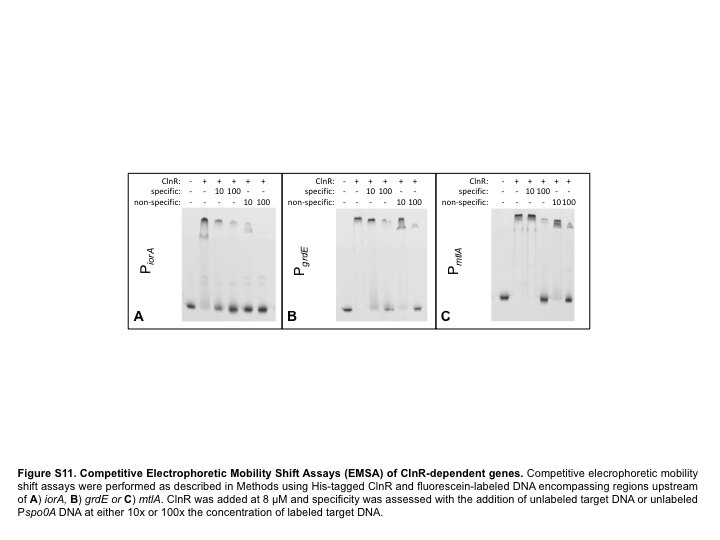

Supplement: S11 Fig — Competitive elecrophoretic mobility shift assays were performed as described in Methods using His-tagged ClnR and fluorescein-labeled DNA encompassing regions upstream of A) iorA, B) grdE or C) mtlA. ClnR was added at 8 μM and specificity was assessed with the addition of unlabeled target DNA or unlabeled Pspo0A DNA at either 10x or 100x the concentration of labeled target DNA. (TIFF) [file ppat.1007153.s011.tiff]
