# Supplementary material for: The C. difficile clnRAB operon initiates adaptations to the host environment in response to LL-37
Source: PLoS Pathog. 2018 Aug 20;14(8):e1007153. doi: 10.1371/journal.ppat.1007153 (PMC6117091; doi:10.1371/journal.ppat.1007153)
Supplement: S1 Table — (PDF) [file ppat.1007153.s012.pdf]

Table S1. Genes differentially expressed in LL-37

Gene[a]	Fold change in LL-37[b]	Product	COG[c]	*p*[d]
**Induced in LL-37**				
CD630_16180 (clnA)	202.99	ABC-type transport system multidrug-family ATP-binding protein	V	0.023
CD630_16190 (clnB)	192.16	ABC-type transport system multidrug-family permease	-	0.001
CD630_16170 (clnR)	186.82	Transcriptional regulator GntR family	K	0.005
CD630_16100	64.45	conserved hypothetical protein	-	4.2E-04
CD630_16110	34.77	conserved hypothetical protein	-	0.028
CD630_16090	30.62	conserved hypothetical protein	-	0.030
CD630_16070	30.25	ABC-type transport system multidrug-family ATP-binding protein	V	0.011
CD630_12400 (vanZ)	28.43	Teicoplanin resistance protein	V	0.009
CD630_23410 (abfD)	14.81	Gamma-aminobutyrate metabolism dehydratase/isomerase	Q	0.005
CD630_23820	13.76	putative pyridoxal phosphate-dependent transferase	E	0.051
CD630_23810 (iorA)	13.66	Indole pyruvate ferredoxin/flavodoxin oxidoreductase	C	0.007
CD630_23390 (cat2)	13.27	4-hydroxybutyrate CoA transferase	C	0.016
CD630_23400	13.14	uncharacterised protein	-	0.053
CD630_23380 (4hbD)	12.95	4-hydroxybutyrate dehydrogenase	C	0.012
CD630_23420 (sucD)	12.71	Succinate-semialdehyde dehydrogenase	C	0.009
CD630_23430 (cat1)	11.85	Succinyl-CoA:coenzyme A transferase	C	0.007
CD630_23440	10.85	putative membrane protein (butyrate conversion)	R	0.005
CD630_23800 (iorB)	10.81	Indole pyruvate ferredoxin/flavodoxin oxidoreductase	C	0.010
CD630_12382	5.60	Fragment of conserved hypothetical protein	-	0.005
CD630_05500	5.52	putative membrane protein	-	0.053
CD630_18870 (csfU)	5.34	Extracytoplasmic function (ECF) sigma factor	K	0.002
CD630_05490	4.74	conserved hypothetical protein	-	0.049
CD630_18890	4.54	ABC-type transport system multidrug-family ATP-binding protein	V	1.9E-04
CD630_25560	4.24	PTS system fructose/mannitol-family IIAB component	GT	0.019
CD630_16990 (ribE)	4.23	Riboflavin synthase alpha subunit	H	0.035
CD630_04900	4.13	putative sugar-phosphate dehydrogenase	ER	0.049
CD630_04980	4.13	putative cell-division FtsK/SpoIIIE-family protein Tn5397 CTn3-Orf21	D	0.012
CD630_04960	4.13	putative conjugative transposon protein DUF961 family Tn5397 CTn3-Orf23	-	0.039
CD630_33740	3.92	putative conjugative transposon protein Tn916-like CTn7-Orf8	-	0.011
CD630_04970	3.86	putative conjugative transposon protein DUF961 family Tn5397 CTn3-Orf22	-	0.008
CD630_18880 (rsiU)	3.69	Extracytoplasmic function (ECF) anti-sigma factor	-	0.004
CD630_17000 (ribD)	3.65	Riboflavin biosynthesis protein	H	0.018
CD630_05000	3.61	putative antirestriction protein Tn5397 CTn3-Orf18	-	0.012
CD630_18900	3.60	ABC-type transport system multidrug-family permease	-	0.014
CD630_10590 (thlA1)	3.56	Acetoacetyl-CoA thiolase 1	I	0.037
CD630_05060	3.54	Reverse transcriptase/maturase/endonuclease Group II intron	V	0.001
CD630_10580 (hbd)	3.51	3-hydroxybutyryl-CoA dehydrogenase	I	0.045
CD630_04670	3.43	putative hydrolase HAD superfamily subfamily IIB	HR	4.2E-05
CD630_04780 (spaF)	3.43	ABC-type transport system lantibiotic/multidrug-family ATP-binding protein	V	0.015
CD630_33730 (mgtA)	3.39	Magnesium-transporting ATPase P-type Tn916-like CTn7-Orf7	P	0.044
CD630_03580	3.38	putative conjugative transposon protein Tn916-like CTn1-Orf3	-	0.024
CD630_05102	3.35	Fragment of putative conjugative transposon protein Tn5397 CTn3-Orf5	-	6.0E-05
CD630_05101	3.31	putative conjugative transposon protein Tn5397 CTn3-Orf8	-	0.001
CD630_15510 (hisH)	3.29	Imidazole glycerol phosphate synthase subunit	E	0.001
CD630_05103	3.27	putative conjugative transposon protein Tn5397 CTn3-Orf4	-	0.028
CD630_02910	3.25	putative peptidase M20A family	E	0.006
CD630_10560 (etfA3)	3.22	Electron transfer flavoprotein subunit alpha	C	0.027
CD630_16120	3.18	putative amidohydrolase	Q	0.007
CD630_23790 (buk2)	3.13	Butyrate kinase	C	0.013
CD630_15500 (hisB)	3.12	Imidazoleglycerol-phosphate dehydratase	E	0.001
CD630_15540 (hisI)	3.10	Histidine biosynthesis bifunctional protein	E	0.018

CD630_10550 (etfB3)	3.07	Electron transfer flavoproteins subunit beta	C	0.023
CD630_05100	3.07	putative RNA polymerase sigma factor Tn5397 CTn3-Orf7	K	0.001
CD630_18020	3.05	putative hydrolase metallo-beta-lactamase superfamily	R	0.036
CD630_08530 (oppB)	3.02	ABC-type transport system oligopeptide-family permease	EP	0.041
CD630_10570 (crt2)	2.97	3-hydroxybutyryl-CoA dehydratase (Crotonase)	I	0.034
CD630_14240	2.97	conserved hypothetical protein	-	0.001
CD630_05770	2.96	conserved hypothetical protein	R	0.008
CD630_33750 (mgtC)	2.96	Magnesium-transporting ATPase protein Tn916-like CTn7-Orf10	S	0.003
CD630_21640 (ldh)	2.95	L-lactate dehydrogenase	C	0.004
CD630_10540 (bcd2)	2.94	Butyryl-CoA dehydrogenase	I	0.017
CD630_15470 (hisZ)	2.92	ATP phosphoribosyltransferase regulatory subunit	E	0.044
CD630_20140 (ilvD)	2.90	Dihydroxy-acid dehydratase	EG	7.2E-05
CD630_08570 (oppF)	2.88	Fragment of ABC-type transport system oligopeptide-family ATP-binding protein	-	0.029
CD630_15490 (hisC)	2.86	Histidinol-phosphate aminotransferase	E	0.047
CD630_15520 (hisA)	2.80	1-(5-phosphoribosyl)-5-[(5-phosphoribosylamino)m ethylideneamino]imidazole-4-carboxamide isomerase	E	0.044
CD630_16590	2.69	Cation-transporting ATPase	P	0.003
CD630_20270	2.69	N-carbamoyl-L-amino acid hydrolase	E	0.047
CD630_33911	2.66	conserved hypothetical protein	-	0.021
CD630_16310 (sodA)	2.59	spore coat protein-superoxide dismutase (Mn)	P	0.007
CD630_03830	2.58	putative cell-division FtsK/SpoIIIE-family protein Tn916-like CTn1-Orf28	D	0.009
CD630_10860	2.54	putative peptidase M20D family	R	0.033
CD630_21930 (cwp24)	2.54	putative cell wall-binding protein	-	0.047
CD630_20910	2.49	putative xanthine/uracil permease	F	0.010
CD630_17880	2.48	putative membrane protein	-	0.027
CD630_16740	2.47	putative NADPH-dependent FMN reductase	R	0.005
CD630_22330 (asrA)	2.43	Anaerobic sulfite reductase subunit A	C	0.043
CD630_17021 (thiS)	2.41	Thiamine biosynthesis protein	H	0.011
CD630_20280 (racX)	2.39	putative aspartate racemase	M	1.2E-04
CD630_27090	2.37	putative oxidoreductase	I	0.010
CD630_09260	2.36	putative phage protein	-	0.050
CD630_13900	2.35	conserved hypothetical protein DUF819 family	S	2.8E-06
CD630_21270	2.34	putative exported protein	-	0.042
CD630_27840 (cwp6)	2.30	putative N-acetylmuramoyl-L-alanineamidase autolysin	M	0.006
CD630_23090	2.29	conserved hypothetical protein	-	0.014
CD630_04800 (spaG)	2.29	ABC-type transport system lantibiotic/multidrug-family permease	S	0.037
CD630_04890	2.26	putative phosphoribosylaminoimidazole-succinocarb oxamide synthetase	F	0.001
CD630_17151	2.24	conserved hypothetical protein	-	0.016
CD630_15100	2.24	conserved hypothetical protein	-	0.006
CD630_07290 (gcvH)	2.23	Glycine cleavage system H protein	E	0.044
CD630_20751	2.21	conserved hypothetical protein	-	0.018
CD630_16130 (cotA)	2.18	spore coat assembly protein	-	0.022
CD630_10980	2.17	Two-component sensor histidine kinase Tn1549-like CTn4-Orf27	T	4.3E-04
CD630_10850	2.17	putative membrane protein	E	0.015
CD630_15560	2.16	putative polysaccharide deacetylase	G	0.001
CD630_15120 (panC)	2.15	Pantothenate synthetase	H	0.018
CD630_16200	2.15	Transporter Major Facilitator Superfamily (MFS)	G	2.6E-04
CD630_33790	2.14	putative conjugative transposon proteinTn916-like CTn7-Orf15	-	0.006
CD630_29620	2.13	conserved hypothetical protein	-	2.8E-04
CD630_04440 (ortB)	2.09	2-amino-4-ketopentanoate thiolase beta subunit	E	0.013
CD630_17170	2.07	uncharacterised protein	S	0.038
CD630_27250	2.07	putative monogalactosyldiacylglycerol synthase	M	0.020
CD630_25171	2.04	putative phage protein	-	0.001
CD630_19440	2.03	Fragment of conserved hypothetical protein	-	1.4E-04
CD630_26820 (pfo)	2.03	Pyruvate-ferredoxin oxidoreductase	C	0.021
CD630_15660 (ilvB)	2.01	Acetolactate synthase large subunit	EH	0.040
CD630_19670	2.01	uncharacterised protein	-	2.3E-04
**Reduced in LL-37**				
CD630_23310 (mtlD)	0.12	Mannitol-1-phosphate 5-dehydrogenase	G	4.4E-04

CD630_26140	0.12	uncharacterised protein DegV family	S	0.004
CD630_23320 (mtlF)	0.12	PTS system mannitol-specific EIIA component	G	0.001
CD630_23330 (mtlR)	0.14	Transcription antiterminator PTS operon regulator	K	2.3E-04
CD630_23340 (mtlA)	0.15	PTS system mannitol-specific IICB component	G	2.1E-05
CD630_19120 (eutA)	0.20	Ethanolamine reactivating factor for ammonialyase eutBC	E	0.018
CD630_19150 (eutL)	0.21	Ethanolamine carboxysome structural protein	E	0.003
CD630_31750 (cggR)	0.22	Transcriptional regulator SorC family	K	0.049
CD630_01631	0.22	conserved hypothetical protein	-	4.8E-04
CD630_09950 (serA)	0.23	putative D-3-phosphoglycerate dehydrogenase	HE	0.007
CD630_24291	0.23	putative 4Fe-4S ferredoxin iron-sulfur binding domain protein	C	0.019
CD630_09080	0.23	putative phage protein	-	7.7E-06
CD630_09960	0.24	conserved hypothetical protein	S	0.008
CD630_30270	0.25	PTS system glucose-like IIA component	G	0.021
CD630_19220 (eutN)	0.25	Ethanolamine carboxysome structural protein	QC	0.016
CD630_20160	0.26	conserved hypothetical protein	-	0.034
23S_rRNA	0.27	23S ribosomal RNA	-	0.026
CD630_09360	0.28	putative phage endodeoxyribonuclease RusA-like	L	0.007
CD630_29341	0.29	putative phage protein	-	0.049
CD630_29470	0.30	putative phage protein	-	0.020
CD630_29320	0.30	putative phage protein	-	0.035
CD630_28780 (fhuD)	0.30	ABC-type transport system ferrichrome-specific extracellular solute-binding protein	P	0.004
CD630_14890 (metN)	0.32	ABC-type transport system methionine-specific ATP-binding protein	P	0.035
CD630_14900 (met I)	0.32	ABC-type transport system methionine-specific permease	P	0.034
CD630_16632	0.32	conserved hypothetical protein	-	0.018
CD630_17452	0.34	conserved hypothetical protein	R	0.026
CD630_21710	0.34	Fragment of putative sodium:dicarboxylate symporter	-	0.009
CD630_31360 (bglA7)	0.35	6-phospho-beta-glucosidase	G	0.001
CD630_26640 (murE)	0.35	UDP-N-acetylmuramyl-tripeptide synthetase	M	2.4E-04
CD630_28770 (fhuB)	0.35	ABC-type transport system ferrichrome-specific permease	P	1.5E-04
CD630_36010	0.35	D-alanyl-D-alanine carboxypeptidase M15 family	M	0.013
CD630_31000	0.36	putative C4-dicarboxylate anaerobic carrier Dcu family	S	0.046
CD630_18470	0.36	putative conjugative transposon protein Tn1549-like CTn5-Orf3	-	0.001
CD630_31150 (bglA4)	0.37	6-phospho-beta-glucosidase	G	0.003
CD630_13610	0.37	putative phage protein	-	0.002
CD630_32570	0.37	putative polysaccharide deacetylase	G	0.016
CD630_30340	0.38	Transcriptional regulator TrmB family	K	0.013
CD630_27491	0.38	Autoinducer prepeptide	-	2.5E-05
CD630_25150	0.38	putative L-aspartate-beta-decarboxylase	E	0.043
CD630_27640	0.38	putative hydrolase HAD superfamily IIB subfamily	R	0.003
CD630_26710	0.38	ABC-type transport system ATP-binding protein putative oligopeptide transport system	EP	0.028
CD630_33440	0.39	putative cell-division FtsK/SpoIIIE-family protein Tn916-like CTn6-Orf22	D	0.007
CD630_09340	0.39	putative phage protein	-	0.007
CD630_35370 (phnH)	0.39	putative phosphonate metabolism protein	P	0.007
CD630_31370 (bglF5)	0.39	PTS system beta-glucoside-specific IIAB component	G	0.022
CD630_30360	0.39	Transporter Major Facilitator Superfamily (MFS)	E	0.022
CD630_08450	0.40	putative nuclease	-	0.034
CD630_22010	0.40	Transporter Major Facilitator Superfamily (MFS)	G	0.045
CD630_25090	0.40	putative glycoside hydrolase family 4	G	0.016
CD630_23710 (nadB)	0.40	L-aspartate oxidase (Quinolinate synthetase B)	H	4.3E-04
CD630_23720 (nadA)	0.40	Quinolinate synthetase A	H	0.003
CD630_13640	0.40	putative phage XkdM-like protein	-	0.001
CD630_19180 (eutK)	0.40	Ethanolamine carboxysome strutural protein	QC	0.039
CD630_11540	0.40	Transcriptional regulator PadR family	K	0.025
CD630_03270 (cbiO)	0.40	ABC-type transport system cobalt-specific ATP-binding protein	P	0.003
CD630_05790	0.40	Transcriptional regulator TetR family	K	0.027
CD630_30990	0.40	putative amidohydrolase M20D family	R	0.031

CD630_03260 (cbiQ1)	0.41	ABC-type transport system cobalt-specific permease	P	0.002
CD630_18860	0.41	Transcriptional regulator PadR family	K	0.014
CD630_30720	0.41	conserved hypothetical protein	S	0.019
CD630_03140	0.41	putative membrane protein	S	0.018
CD630_26860	0.41	putative membrane protein	-	0.009
CD630_24620 (grpE)	0.41	HSP-70 cofactor	O	4.5E-04
CD630_29420	0.41	putative phage resolvase/integrase	-	1.4E-04
CD630_11261	0.42	Transcriptional regulator HTH-type	K	0.004
CD630_29400	0.42	putative phage protein	-	0.043
CD630_17540	0.42	ABC-type transport system multidrug-family permease	-	0.018
CD630_03240 (cbiM)	0.42	Cobalamin biosynthesis protein	P	0.009
CD630_23700 (nadC)	0.42	Nicotinate-nucleotide pyrophosphorylase	H	0.002
CD630_06140	0.43	conserved hypothetical protein	-	0.027
CD630_26700	0.43	ABC-type transport system ATP-binding protein putative oligopeptide transport system	E	0.025
CD630_01060 (cwlD)	0.43	Germination-specific N-acetylmuramoyl-L-alanineamidase Autolysin	M	0.009
CD630_26650	0.43	Transcriptional regulator AraC family	K	0.014
CD630_03901	0.43	conserved hypothetical protein	-	0.021
CD630_25160 (ansB)	0.44	L-asparaginase	EJ	0.034
CD630_29360	0.44	putative phage protein	-	0.007
CD630_05670	0.44	uncharacterised protein DegV family	S	0.033
CD630_01250	0.44	putative cell wall endopeptidase	M	0.046
CD630_04090	0.44	putative replication initiation protein Tn1549-like CTn2-Orf2	-	0.029
CD630_29440	0.44	putative phage essential recombination functionprotein	-	0.040
CD630_12700	0.45	Two-component sensor histidine kinase	T	0.001
CD630_30260	0.45	conserved hypothetical protein	TK	0.005
CD630_11710 (etfB4)	0.45	Electron transfer flavoprotein subunit alpha	C	0.008
CD630_29430	0.45	putative phage replication protein	L	0.010
CD630_09170	0.45	putative phage recombination protein Bet	-	6.1E-05
CD630_06240	0.45	putative transcriptional regulator activator	S	0.003
CD630_29710 (bioY)	0.45	Biotin synthase	R	0.012
CD630_01930 (groS)	0.46	chaperonin	O	0.003
CD630_20451	0.46	conserved hypothetical protein	-	1.9E-04
CD630_13450	0.46	Transcriptional regulator PadR family	K	0.002
CD630_26870	0.46	conserved hypothetical protein	-	0.007
CD630_29490	0.46	Transcriptional regulator Phage-type	-	0.005
CD630_05780	0.47	Transporter Major Facilitator Superfamily (MFS)	-	0.004
CD630_18270	0.47	Transcriptional regulator MarR family	K	0.033
CD630_30250	0.47	putative ferredoxin iron-sulphur domain-containing protein	C	0.007
CD630_32090	0.47	Transcriptional regulator PadR family	K	0.006
CD630_00470 (ispD)	0.48	2-C-methyl-D-erythritol 4-phosphatecytidylyltransferase	I	0.029
CD630_21430	0.48	Transcriptional regulator HTH-type	-	0.020
CD630_24610 (dnaK)	0.48	Chaperone protein dnaK (Heat shock protein 70)	O	0.009
CD630_25110	0.48	Transcription antiterminator PTS operon regulator	K	0.026
CD630_32100	0.48	conserved hypothetical protein	E	0.030
CD630_32620 (pstA)	0.48	ABC-type transport system phosphate-specific permease	P	0.045
CD630_25100	0.48	PTS system glucose-like IIBC component	G	0.043
CD630_27880	0.48	putative membrane protein GtrA family	S	0.004
CD630_13720	0.48	putative phage XkdT-like protein	-	2.2E-06
CD630_19210	0.48	putative ethanolamine utilization protein	-	0.017
CD630_10280	0.48	putative signaling protein	TK	0.030
CD630_21510	0.48	putative membrane protein DUF819 family	S	0.006
CD630_23300 (xpt)	0.49	Xanthine phosphoribosyltransferase (XPRTase)	F	0.036
CD630_15800 (hom2)	0.49	Homoserine dehydrogenase	E	0.010
CD630_24630 (hrcA)	0.49	Transcriptional regulator Heat-inducibler epressor	K	0.013
CD630_32220 (sdaB)	0.49	L-serine dehydratase	E	0.005
CD630_27500 (agrB)	0.49	Accessory gene regulator	OTK	0.001
CD630_32600 (phoU)	0.49	Phosphate uptake regulator	P	0.001
CD630_13460	0.50	conserved hypothetical protein DUF1700	S	0.002

CD630_26691	0.50	putative Na(+)/H(+) antiporter	P	0.009
CD630_29330	0.50	Hypothetical protein	J	0.010
CD630_03250 (cbiN)	0.50	ABC-type transport system cobalt-specific extracellular solute-binding protein	P	0.031

[a]Gene accession numbers are given for strain 630.
[b]Ratio of no LL-37/with LL-37 as determined by RNA sequencing analysis of 630Δ*erm* grown in BHIS alone or BHIS supplemented with 2 μg/ml LL-37 as described in Methods. Genes are included in this list if they had ≥ 2-fold increase or decrease in expression and a *P* value ≤ 0.05 by Student's two-tailed t-test.
[c]COG (classification of gene) designations are based on the 2014 COG database. Letter designations correspond to the categories listed in the table below.
[d]*P*-values determined by Student's two-tailed *t*-test.

A	RNA processing and modification
B	Chromatin Structure and dynamics
C	Energy production and conversion
D	Cell cycle control and mitosis
E	Amino Acid metabolism and transport
F	Nucleotide metabolism and transport
G	Carbohydrate metabolism and transport
H	Coenzyme metabolism
I	Lipid metabolism
J	Translation
K	Transcription
L	Replication and repair
M	Cell wall/membrane/envelop biogenesis
N	Cell motility
O	Post-translational modification, protein turnover, chaperone functions
P	Inorganic ion transport and metabolism
Q	Secondary Structure
T	Signal Transduction
U	Intracellular trafficking and secretion
Y	Nuclear structure
Z	Cytoskeleton
R	General Functional Prediction only
S	Function Unknown
-	Unassigned
