# Supplementary material for: The C. difficile clnRAB operon initiates adaptations to the host environment in response to LL-37
Source: PLoS Pathog. 2018 Aug 20;14(8):e1007153. doi: 10.1371/journal.ppat.1007153 (PMC6117091; doi:10.1371/journal.ppat.1007153)
Supplement: S2 Table — (PDF) [file ppat.1007153.s013.pdf]

Table S2. LL-37 MIC and MBC values for *clnR* and *clnAB* mutants

	MIC[a]	MBC[b]
630Δ*erm*	15	20
clnR	15	30
clnAB	15	20

[a] Minimum inhibitory concentration of LL-37 (µg/ml).
[b] Minimum bactericidal concentration of LL-37 (µg/ml).
