# Supplementary material for: The C. difficile clnRAB operon initiates adaptations to the host environment in response to LL-37
Source: PLoS Pathog. 2018 Aug 20;14(8):e1007153. doi: 10.1371/journal.ppat.1007153 (PMC6117091; doi:10.1371/journal.ppat.1007153)
Supplement: S3 Table — (PDF) [file ppat.1007153.s014.pdf]

**Table S3. MIC values for *clnR* and *clnAB* mutants in various antimicrobials**

| | Van[a] | Amp | PmB | Nis |
|---|---|---|---|---|
| 630Δ*erm* | 1 | 4 | 500 | 360 |
| *clnR* | 1 | 4 | 500 | 360 |
| *clnAB* | 1 | 4 | 500 | 360 |

[a]Values shown are μg/ml. Van: vancomycin, Amp: ampicillin, PmB: polymyxin B, Nis: nisin.
