# Supplementary material for: The C. difficile clnRAB operon initiates adaptations to the host environment in response to LL-37
Source: PLoS Pathog. 2018 Aug 20;14(8):e1007153. doi: 10.1371/journal.ppat.1007153 (PMC6117091; doi:10.1371/journal.ppat.1007153)
Supplement: S4 Table — (PDF) [file ppat.1007153.s015.pdf]

Table S4. Genes differentially expressed in a *clnR* mutant in the presence/absence of LL-37

Gene[a]	*clnR*/ WT[b]	Product	COG[c]	*p*[d]
**ClnR activates (without LL-37)**				
CD630_16632	0.22	conserved hypothetical protein	-	0.003
CD630_23030	0.22	Fragment of putative phage transcriptional repressor	-	0.003
CD630_35370 (phnH)	0.22	putative phosphonate metabolism protein	P	9.0E-05
CD630_19280	0.22	putative membrane protein	D	9.0E-05
CD630_29341	0.23	putative phage protein	-	0.009
CD630_01631	0.24	conserved hypothetical protein	-	0.013
CD630_13700	0.25	putative phage XkdS-like protein	-	0.018
CD630_32770	0.26	PTS system mannose/fructose/sorbose IIC component	G	0.004
CD630_29330	0.32	Hypothetical protein	J	0.005
CD630_04090	0.33	putative replication initiation protein Tn1549-like CTn2-Orf2	-	0.032
CD630_00410	0.33	PTS system galactitol-specific IIA component	GT	2.7E-04
CD630_11980 (spoIIIAG)	0.33	Stage III sporulation protein AG	-	0.001
CD630_07400	0.35	putative pyridoxal phosphate-dependent aminotransferase	E	0.008
CD630_07420	0.37	putative ethanolamine transporter	E	0.003
CD630_36361	0.38	conserved hypothetical protein	-	0.015
CD630_29370	0.39	putative phage protein	-	0.011
CD630_01350	0.41	PTS system lactose/cellobiose-family IIA component	G	0.032
CD630_23260	0.41	PTS system fructose/mannitol family IIBc omponent	G	0.001
CD630_24170	0.42	PTS system Sorbitol-like IIB component	G	0.003
CD630_26870	0.43	conserved hypothetical protein	-	0.002
CD630_01981	0.43	conserved hypothetical protein	-	0.042
CD630_02850	0.44	PTS system mannose/fructose/sorbose IIB component	G	0.001
CD630_29490	0.44	Transcriptional regulator Phage-type	-	3.1E-04
CD630_13630	0.45	putative phage XkdK-like protein	-	0.016
CD630_29360	0.45	putative phage protein	-	0.009
CD630_16490	0.45	ABC-type transport system iron-family ATP-binding protein	P	0.022
CD630_21970	0.46	putative ferredoxin/flavodoxin oxidoreductase	C	0.017
CD630_12332	0.46	conserved hypothetical protein	-	0.034
CD630_26570	0.49	conserved hypothetical protein	-	0.006
CD630_04200	0.49	putative cell surface protein Tn1549-like CTn2-Orf15	R	0.006
CD630_21021	0.49	conserved hypothetical protein	-	0.006
CD630_04880 (orr)	0.50	putative small multidrug resistance SugE-like protein	P	0.041
**ClnR represses (without LL-37)**				
CD630_16180 (clnA)	57.26	ABC-type transport system multidrug-family ATP-binding protein	V	0.021
CD630_16190 (clnB)	53.69	ABC-type transport system multidrug-family permease	-	0.001
CD630_12400 (vanZ)	21.50	Teicoplanin resistance protein	V	0.017
CD630_33370	3.37	putative membrane protein Tn916-like CTn6-Orf14	B	0.006
CD630_07970	2.96	putative pyruvate carboxyltransferase	E	0.014
CD630_16310 (sodA)	2.70	spore coat protein-superoxide dismutase	P	4.3E-04
CD630_01820	2.47	conserved hypothetical protein	-	0.017
CD630_18840	2.43	conserved hypothetical protein	-	0.042
CD630_22180 (int2)	2.43	Fragment of putative integrase	-	0.042
CD630_03710	2.43	putative conjugative transposon protein Tn916-like CTn1-Orf16	-	0.042
CD630_15111	2.26	conserved hypothetical protein	-	0.037
CD630_16680	2.13	putative membrane protein	-	3.4E-06
CD630_27340	2.09	putative Na+/H+ antiporter NhaC family	C	0.001
CD630_12450	2.08	conserved hypothetical protein	-	0.032
**ClnR activates (with LL-37)**				
CD630_23540 (grdE)	0.06	Betaine reductase component B subunit	-	0.001
CD630_23520 (grdA)	0.07	Glycine reductase complex selenoprotein A (selenocysteine)	-	1.5E-04
CD630_02850	0.07	PTS system mannose/fructose/sorbose IIB component	G	0.002
CD630_02840	0.08	PTS system mannose/fructose/sorbose IIA component	G	0.005
CD630_23550 (trxA2)	0.09	Thioredoxin 2	O	0.005
CD630_23260	0.09	PTS system fructose/mannitol family IIB component	G	0.008
CD630_02880	0.10	PTS system mannose/fructose/sorbose IIC component	G	3.3E-04
CD630_16080	0.13	ABC-type transport system multidrug-family permease	-	0.003

CD630_02890	0.13	PTS system mannose/fructose/sorbose IID component	G	0.002
CD630_02860	0.13	PTS system mannose/fructose/sorbose IIA component	G	0.014
CD630_16060	0.13	Transcriptional regulator GntR family	K	0.001
CD630_16100	0.13	conserved hypothetical protein	-	0.001
CD630_16110	0.14	conserved hypothetical protein	-	0.003
CD630_02870	0.14	PTS system mannose/fructose/sorbose IIB component	G	0.002
CD630_23570 (grdX)	0.14	putative glycine reductase complex component	-	0.017
CD630_16090	0.15	conserved hypothetical protein	-	0.004
CD630_23560 (trxB3)	0.15	Thioredoxin reductase 3	O	0.018
CD630_23490 (grdC)	0.16	Glycine reductase complex component C	I	0.007
CD630_16070	0.16	ABC-type transport system multidrug-family ATP-binding protein	V	0.003
CD630_20751	0.18	conserved hypothetical protein	-	0.002
CD630_29290	0.18	putative phage protein	-	0.004
CD630_04200	0.20	putative cell surface protein Tn1549-like CTn2-Orf15	R	0.001
CD630_23240	0.21	putative sugar-phosphate dehydrogenase	ER	0.050
CD630_33740	0.21	putative conjugative transposon protein Tn916-like CTn7-Orf8	-	6.2E-05
CD630_23030	0.21	Fragment of putative phage transcriptional repressor	-	0.006
CD630_26000 (cstA)	0.22	Carbon starvation protein	T	1.4E-05
CD630_23810 (iorA)	0.22	Indole pyruvate ferredoxin/flavodoxin oxidoreductase	C	8.8E-05
CD630_04670	0.24	putative hydrolase HAD superfamily subfamily IIB	HR	0.001
CD630_23800 (iorB)	0.24	Indole pyruvate ferredoxin/flavodoxin oxidoreductase	C	4.0E-04
CD630_25990	0.24	putative transcriptional regulator	J	0.040
CD630_02900	0.25	conserved hypothetical protein	-	0.002
CD630_23820	0.26	putative pyridoxal phosphate-dependent transferase	E	0.002
CD630_29410	0.27	putative phage single-strand DNA-binding protein	L	0.037
CD630_16180 (clnA)	0.27	ABC-type transport system multidrug-family ATP-binding protein	V	0.005
CD630_33750 (mgtC)	0.27	Magnesium-transporting ATPase protein Tn916-like CTn7-Orf10	S	0.002
CD630_16190 (clnB)	0.27	ABC-type transport system multidrug-family permease	-	0.011
CD630_33730 (mgtA)	0.27	Magnesium-transporting ATPase P-type Tn916-like CTn7-Orf7	P	0.001
CD630_00330	0.28	putative glycoside hydrolase	G	0.048
CD630_07400	0.28	putative pyridoxal phosphate-dependent aminotransferase	E	0.045
CD630_05490	0.28	conserved hypothetical protein	-	0.001
CD630_33741	0.29	putative conjugative transposon protein Tn916-like CTn7-Orf9	-	0.002
CD630_28130 (garR)	0.30	Tartronate semialdehyde reductase	I	0.042
CD630_29370	0.31	putative phage protein	-	0.001
CD630_00220 (fusA1)	0.31	Elongation factor G	J	0.013
CD630_18570	0.31	putative cell wall hydrolase Tn1549-like CTn5-Orf13	M	0.038
CD630_18880 (rsiU)	0.32	Extracytoplasmic function (ECF) anti-sigma factor	-	0.004
CD630_05000	0.32	putative antirestriction protein Tn5397 CTn3-Orf18	V	0.006
CD630_17970	0.34	Coenzyme A disulfide reductase	R	0.037
CD630_01350	0.34	PTS system lactose/cellobiose-family IIA component	G	0.048
CD630_04960	0.34	putative conjugative transposon protein DUF961 family Tn5397 CTn3-Orf23	-	0.007
CD630_23480 (grdD)	0.34	Glycine reductase complex component C	I	0.015
CD630_04980	0.34	putative cell-division FtsK/SpoIIIE-family protein Tn5397 CTn3-Orf21	D	0.001
CD630_17000 (ribD)	0.35	Riboflavin biosynthesis protein	H	0.014
CD630_28620	0.36	putative peptidase M19 family	E	0.016
CD630_18560	0.36	putative hydrolase Tn1549-like CTn5-Orf12	U	0.002
CD630_15970	0.36	conserved hypothetical protein	-	3.0E-04
CD630_28240	0.36	Fragment of putative membrane protein	-	0.020
CD630_33721	0.36	putative conjugative transposon protein Tn916-like CTn7-Orf5	-	0.004
CD630_04970	0.36	putative conjugative transposon protein DUF961 family Tn5397 CTn3-Orf22	-	0.008
CD630_05103	0.37	putative conjugative transposon proteinTn5397%2CCTn3-Orf4	-	0.013
CD630_04990	0.38	putative replication initiation factor Tn5397 CTn3-Orf20	L	0.001
CD630_22820	0.39	PTS system fructose/mannitol family IIA component	GT	0.024
CD630_04991	0.39	putative conjugative transposon protein Tn5397 CTn3-Orf19	-	0.005
CD630_02910	0.40	putative peptidase M20A family	E	0.009
CD630_16980 (ribBA)	0.40	GTP cyclohydrolase-2 Riboflavin biosynthesis protein	H	0.012
CD630_05100	0.40	putative RNA polymerase sigma factor Tn5397 CTn3-Orf7	K	0.001
CD630_17960	0.40	putative nitrite and sulfite reductase subunit	C	0.044

CD630_05102	0.40	Fragment of putative conjugative transposon protein Tn5397 CTn3-Orf5	-	0.002
CD630_05060	0.41	Reverse transcriptase/maturase/endonuclease Group II intron	V	0.001
CD630_33722	0.41	putative conjugative transposon protein Tn916-like CTn7-Orf6	-	0.002
CD630_23790 (buk2)	0.41	Butyrate kinase	C	2.5E-04
CD630_18870 (csfU)	0.41	Extracytoplasmic function (ECF) sigma factor	K	0.001
CD630_28280	0.41	putative pyridoxal phosphate-dependent transferase	E	0.029
CD630_05101	0.41	putative conjugative transposon protein Tn5397 CTn3-Orf8	-	1.6E-04
CD630_07390	0.42	putative exported protein	-	1.0E-04
CD630_15431	0.43	conserved hypothetical protein	-	0.015
CD630_20750 (pbuX)	0.43	putative xanthine permease	F	0.001
CD630_28110	0.43	conserved hypothetical protein	S	0.010
CD630_08650	0.44	putative ADP-ribose binding protein	R	0.013
CD630_06790	0.45	conserved hypothetical protein	R	0.018
CD630_04890	0.45	putative phosphoribosylaminoimidazole-succinocarboxamide synthetase	F	0.001
CD630_23590	0.46	putative hydrolase HAD superfamily subfamily IIB	R	2.9E-04
CD630_28590	0.46	putative D-aminoacylase	Q	0.018
CD630_18890	0.47	ABC-type transport system multidrug-family ATP-binding protein	V	0.004
CD630_06770 (csfT)	0.47	Extracytoplasmic function (ECF) sigma factor	K	0.001
CD630_21600	0.47	putative membrane protein	V	0.019
CD630_16220	0.47	peptidase propeptide and ypeb domain protein	S	0.034
CD630_24020	0.47	putative cell wall hydrolase phosphatase-associated protein	M	0.008
CD630_18900	0.47	ABC-type transport system multidrug-family permease	O	0.007
CD630_04400 (cwp27)	0.47	putative cell wall binding protein	-	8.9E-05
CD630_29490	0.48	Transcriptional regulator Phage-type	-	0.004
CD630_27080 (aroE2)	0.48	Shikimate dehydrogenase 2	E	8.6E-05
CD630_09370	0.48	putative phage anti-repressor	K	0.012
CD630_25670	0.49	PTS system mannose-specfic IIB component	G	0.009
CD630_05020	0.49	putative ATPase Tn5397 CTn3-Orf16	L	0.002
CD630_29300	0.49	putative phage anti-repressor protein	K	0.012
CD630_27840 (cwp6)	0.49	putative N-acetylmuramoyl-L-alanineamidase autolysin	M	0.002
CD630_01420	0.49	putative RNA-binding protein	J	0.028
CD630_22490	0.50	putative ATPase	R	0.001
CD630_21910	0.50	putative phosphoesterase	ER	0.008
CD630_23090	0.50	conserved hypothetical protein	-	0.039
**ClnR represses (with LL-37)**				
CD630_26140	7.62	uncharacterised protein DegV family	S	0.017
CD630_06680	4.19	Two-component response regulator	K	0.014
CD630_06690	4.01	Two-component sensor histidine kinase	T	0.011
CD630_27491 (agrD)	3.46	Autoinducer prepeptide	-	0.002
CD630_11920	3.10	Stage III sporulation protein AA	S	0.039
CD630_24291	3.10	putative 4Fe-4S ferredoxin iron-sulfur binding domain protein	C	0.039
CD630_28370	2.74	putative membrane protein	S	0.046
CD630_04370	2.74	Fragment of integrase Tn1549-like CTn2	-	0.046
CD630_09780	2.52	Transcriptional regulator beta-lactams repressor Phage-type	-	0.011
CD630_22290	2.49	putative membrane protein	S	0.049
CD630_10950	2.48	ABC-type transport system multidrug-family permease Tn1549-like CTn4-Orf30	-	0.030
CD630_25150	2.45	putative L-aspartate-beta-decarboxylase	E	0.039
CD630_04640	2.45	putative beta-lactamase-like hydrolase	R	0.018
CD630_26640 (murE)	2.42	UDP-N-acetylmuramyl-tripeptide synthetase	M	0.009
CD630_09950 (serA)	2.40	putative D-3-phosphoglycerate dehydrogenase	HE	0.019
CD630_09960	2.34	conserved hypothetical protein	S	0.015
CD630_21510	2.34	putative membrane protein DUF819 family	S	0.007
CD630_33360	2.32	putative cell wall hydrolase Tn916-like CTn6-Orf13	M	5.0E-04
CD630_01260 (spoIIID)	2.29	Stage III sporulation protein D	-	0.002
CD630_30730	2.24	putative membrane protein	-	0.008
CD630_10970	2.22	ABC-type transport system multidrug-family ATP-binding protein Tn1549-like CTn4-Orf28	V	0.008
CD630_25290	2.18	conserved hypothetical protein	-	0.021
CD630_21710	2.18	Fragment of putative sodium:dicarboxylate symporter	-	0.036

CD630_08220	2.11	ABC-type transport system multidrug-family ATP-binding protein	V	0.016
CD630_26700	2.10	ABC-type transport system ATP-binding protein putative oligopeptide transport system	E	0.031
CD630_25940 (uraA)	2.10	ABC-type transport system uracil-specific permease	F	4.5E-04
CD630_08230	2.05	ABC-type transport system multidrug-family permease	-	0.042
CD630_30230	2.03	conserved hypothetical protein	-	0.034
CD630_23750	2.03	uncharacterised protein	-	0.022

[a]Gene accession numbers are given for strain 630. Gene *CD630_20072 (ermB*) is excluded from these tables because it is an artifact of the insertional disruption used to generate the *clnR* mutant.
[b]Ratio of expression in *clnR* mutant/630Δ*erm*, as determined by RNA sequencing analysis of 630Δ*erm* and *clnR* grown in BHIS alone or BHIS supplemented with 2 μg/ml LL-37 as described in Methods. Fold-change represents the ratio of expression in *clnR*/630Δ*erm* in the condition indicated (with or without LL-37). Genes are included in this list if they had ≥ 2-fold increase or decrease in expression and a *p* value ≤ 0.05 by Student's two-tailed *t*-test.
[c]COG designations are based on the 2014 COG database. Letter designations correspond to the categories listed in the table associated with Table S1.
[d]*p* values determined by Student's two-tailed *t*-test.
