# Supplementary material for: The C. difficile clnRAB operon initiates adaptations to the host environment in response to LL-37
Source: PLoS Pathog. 2018 Aug 20;14(8):e1007153. doi: 10.1371/journal.ppat.1007153 (PMC6117091; doi:10.1371/journal.ppat.1007153)
Supplement: S5 Table — (PDF) [file ppat.1007153.s016.pdf]

Table S1. Genes differentially expressed in LL-37

Gene[a]	Fold change in LL-37[b]	Product	COG[c]	*p*[d]
**Induced in LL-37**				
CD630_16180 (clnA)	202.99	ABC-type transport system multidrug-family ATP-binding protein	V	0.023
CD630_16190 (clnB)	192.16	ABC-type transport system multidrug-family permease	-	0.001
CD630_16170 (clnR)	186.82	Transcriptional regulator GntR family	K	0.005
CD630_16100	64.45	conserved hypothetical protein	-	4.2E-04
CD630_16110	34.77	conserved hypothetical protein	-	0.028
CD630_16090	30.62	conserved hypothetical protein	-	0.030
CD630_16070	30.25	ABC-type transport system multidrug-family ATP-binding protein	V	0.011
CD630_12400 (vanZ)	28.43	Teicoplanin resistance protein	V	0.009
CD630_23410 (abfD)	14.81	Gamma-aminobutyrate metabolism dehydratase/isomerase	Q	0.005
CD630_23820	13.76	putative pyridoxal phosphate-dependent transferase	E	0.051
CD630_23810 (iorA)	13.66	Indole pyruvate ferredoxin/flavodoxin oxidoreductase	C	0.007
CD630_23390 (cat2)	13.27	4-hydroxybutyrate CoA transferase	C	0.016
CD630_23400	13.14	uncharacterised protein	-	0.053
CD630_23380 (4hbD)	12.95	4-hydroxybutyrate dehydrogenase	C	0.012
CD630_23420 (sucD)	12.71	Succinate-semialdehyde dehydrogenase	C	0.009
CD630_23430 (cat1)	11.85	Succinyl-CoA:coenzyme A transferase	C	0.007
CD630_23440	10.85	putative membrane protein (butyrate conversion)	R	0.005
CD630_23800 (iorB)	10.81	Indole pyruvate ferredoxin/flavodoxin oxidoreductase	C	0.010
CD630_12382	5.60	Fragment of conserved hypothetical protein	-	0.005
CD630_05500	5.52	putative membrane protein	-	0.053
CD630_18870 (csfU)	5.34	Extracytoplasmic function (ECF) sigma factor	K	0.002
CD630_05490	4.74	conserved hypothetical protein	-	0.049
CD630_18890	4.54	ABC-type transport system multidrug-family ATP-binding protein	V	1.9E-04
CD630_25560	4.24	PTS system fructose/mannitol-family IIAB component	GT	0.019
CD630_16990 (ribE)	4.23	Riboflavin synthase alpha subunit	H	0.035
CD630_04900	4.13	putative sugar-phosphate dehydrogenase	ER	0.049
CD630_04980	4.13	putative cell-division FtsK/SpoIIIE-family protein Tn5397 CTn3-Orf21	D	0.012
CD630_04960	4.13	putative conjugative transposon protein DUF961 family Tn5397 CTn3-Orf23	-	0.039
CD630_33740	3.92	putative conjugative transposon protein Tn916-like CTn7-Orf8	-	0.011
CD630_04970	3.86	putative conjugative transposon protein DUF961 family Tn5397 CTn3-Orf22	-	0.008
CD630_18880 (rsiU)	3.69	Extracytoplasmic function (ECF) anti-sigma factor	-	0.004
CD630_17000 (ribD)	3.65	Riboflavin biosynthesis protein	H	0.018
CD630_05000	3.61	putative antirestriction protein Tn5397 CTn3-Orf18	-	0.012
CD630_18900	3.60	ABC-type transport system multidrug-family permease	-	0.014
CD630_10590 (thlA1)	3.56	Acetoacetyl-CoA thiolase 1	I	0.037
CD630_05060	3.54	Reverse transcriptase/maturase/endonuclease Group II intron	V	0.001
CD630_10580 (hbd)	3.51	3-hydroxybutyryl-CoA dehydrogenase	I	0.045
CD630_04670	3.43	putative hydrolase HAD superfamily subfamily IIB	HR	4.2E-05
CD630_04780 (spaF)	3.43	ABC-type transport system lantibiotic/multidrug-family ATP-binding protein	V	0.015
CD630_33730 (mgtA)	3.39	Magnesium-transporting ATPase P-type Tn916-like CTn7-Orf7	P	0.044
CD630_03580	3.38	putative conjugative transposon protein Tn916-like CTn1-Orf3	-	0.024
CD630_05102	3.35	Fragment of putative conjugative transposon protein Tn5397 CTn3-Orf5	-	6.0E-05
CD630_05101	3.31	putative conjugative transposon protein Tn5397 CTn3-Orf8	-	0.001
CD630_15510 (hisH)	3.29	Imidazole glycerol phosphate synthase subunit	E	0.001
CD630_05103	3.27	putative conjugative transposon protein Tn5397 CTn3-Orf4	-	0.028
CD630_02910	3.25	putative peptidase M20A family	E	0.006
CD630_10560 (etfA3)	3.22	Electron transfer flavoprotein subunit alpha	C	0.027
CD630_16120	3.18	putative amidohydrolase	Q	0.007
CD630_23790 (buk2)	3.13	Butyrate kinase	C	0.013
CD630_15500 (hisB)	3.12	Imidazoleglycerol-phosphate dehydratase	E	0.001
CD630_15540 (hisI)	3.10	Histidine biosynthesis bifunctional protein	E	0.018

CD630_10550 (etfB3)	3.07	Electron transfer flavoproteins subunit beta	C	0.023
CD630_05100	3.07	putative RNA polymerase sigma factor Tn5397 CTn3-Orf7	K	0.001
CD630_18020	3.05	putative hydrolase metallo-beta-lactamase superfamily	R	0.036
CD630_08530 (oppB)	3.02	ABC-type transport system oligopeptide-family permease	EP	0.041
CD630_10570 (crt2)	2.97	3-hydroxybutyryl-CoA dehydratase (Crotonase)	I	0.034
CD630_14240	2.97	conserved hypothetical protein	-	0.001
CD630_05770	2.96	conserved hypothetical protein	R	0.008
CD630_33750 (mgtC)	2.96	Magnesium-transporting ATPase protein Tn916-like CTn7-Orf10	S	0.003
CD630_21640 (ldh)	2.95	L-lactate dehydrogenase	C	0.004
CD630_10540 (bcd2)	2.94	Butyryl-CoA dehydrogenase	I	0.017
CD630_15470 (hisZ)	2.92	ATP phosphoribosyltransferase regulatory subunit	E	0.044
CD630_20140 (ilvD)	2.90	Dihydroxy-acid dehydratase	EG	7.2E-05
CD630_08570 (oppF)	2.88	Fragment of ABC-type transport system oligopeptide-family ATP-binding protein	-	0.029
CD630_15490 (hisC)	2.86	Histidinol-phosphate aminotransferase	E	0.047
CD630_15520 (hisA)	2.80	1-(5-phosphoribosyl)-5-[(5-phosphoribosylamino)m ethylideneamino]imidazole-4-carboxamide isomerase	E	0.044
CD630_16590	2.69	Cation-transporting ATPase	P	0.003
CD630_20270	2.69	N-carbamoyl-L-amino acid hydrolase	E	0.047
CD630_33911	2.66	conserved hypothetical protein	-	0.021
CD630_16310 (sodA)	2.59	spore coat protein-superoxide dismutase (Mn)	P	0.007
CD630_03830	2.58	putative cell-division FtsK/SpoIIIE-family protein Tn916-like CTn1-Orf28	D	0.009
CD630_10860	2.54	putative peptidase M20D family	R	0.033
CD630_21930 (cwp24)	2.54	putative cell wall-binding protein	-	0.047
CD630_20910	2.49	putative xanthine/uracil permease	F	0.010
CD630_17880	2.48	putative membrane protein	-	0.027
CD630_16740	2.47	putative NADPH-dependent FMN reductase	R	0.005
CD630_22330 (asrA)	2.43	Anaerobic sulfite reductase subunit A	C	0.043
CD630_17021 (thiS)	2.41	Thiamine biosynthesis protein	H	0.011
CD630_20280 (racX)	2.39	putative aspartate racemase	M	1.2E-04
CD630_27090	2.37	putative oxidoreductase	I	0.010
CD630_09260	2.36	putative phage protein	-	0.050
CD630_13900	2.35	conserved hypothetical protein DUF819 family	S	2.8E-06
CD630_21270	2.34	putative exported protein	-	0.042
CD630_27840 (cwp6)	2.30	putative N-acetylmuramoyl-L-alanineamidase autolysin	M	0.006
CD630_23090	2.29	conserved hypothetical protein	-	0.014
CD630_04800 (spaG)	2.29	ABC-type transport system lantibiotic/multidrug-family permease	S	0.037
CD630_04890	2.26	putative phosphoribosylaminoimidazole-succinocarb oxamide synthetase	F	0.001
CD630_17151	2.24	conserved hypothetical protein	-	0.016
CD630_15100	2.24	conserved hypothetical protein	-	0.006
CD630_07290 (gcvH)	2.23	Glycine cleavage system H protein	E	0.044
CD630_20751	2.21	conserved hypothetical protein	-	0.018
CD630_16130 (cotA)	2.18	spore coat assembly protein	-	0.022
CD630_10980	2.17	Two-component sensor histidine kinase Tn1549-like CTn4-Orf27	T	4.3E-04
CD630_10850	2.17	putative membrane protein	E	0.015
CD630_15560	2.16	putative polysaccharide deacetylase	G	0.001
CD630_15120 (panC)	2.15	Pantothenate synthetase	H	0.018
CD630_16200	2.15	Transporter Major Facilitator Superfamily (MFS)	G	2.6E-04
CD630_33790	2.14	putative conjugative transposon proteinTn916-like CTn7-Orf15	-	0.006
CD630_29620	2.13	conserved hypothetical protein	-	2.8E-04
CD630_04440 (ortB)	2.09	2-amino-4-ketopentanoate thiolase beta subunit	E	0.013
CD630_17170	2.07	uncharacterised protein	S	0.038
CD630_27250	2.07	putative monogalactosyldiacylglycerol synthase	M	0.020
CD630_25171	2.04	putative phage protein	-	0.001
CD630_19440	2.03	Fragment of conserved hypothetical protein	-	1.4E-04
CD630_26820 (pfo)	2.03	Pyruvate-ferredoxin oxidoreductase	C	0.021
CD630_15660 (ilvB)	2.01	Acetolactate synthase large subunit	EH	0.040
CD630_19670	2.01	uncharacterised protein	-	2.3E-04
**Reduced in LL-37**				
CD630_23310 (mtlD)	0.12	Mannitol-1-phosphate 5-dehydrogenase	G	4.4E-04

CD630_26140	0.12	uncharacterised protein DegV family	S	0.004
CD630_23320 (mtlF)	0.12	PTS system mannitol-specific EIIA component	G	0.001
CD630_23330 (mtlR)	0.14	Transcription antiterminator PTS operon regulator	K	2.3E-04
CD630_23340 (mtlA)	0.15	PTS system mannitol-specific IICB component	G	2.1E-05
CD630_19120 (eutA)	0.20	Ethanolamine reactivating factor for ammonialyase eutBC	E	0.018
CD630_19150 (eutL)	0.21	Ethanolamine carboxysome structural protein	E	0.003
CD630_31750 (cggR)	0.22	Transcriptional regulator SorC family	K	0.049
CD630_01631	0.22	conserved hypothetical protein	-	4.8E-04
CD630_09950 (serA)	0.23	putative D-3-phosphoglycerate dehydrogenase	HE	0.007
CD630_24291	0.23	putative 4Fe-4S ferredoxin iron-sulfur binding domain protein	C	0.019
CD630_09080	0.23	putative phage protein	-	7.7E-06
CD630_09960	0.24	conserved hypothetical protein	S	0.008
CD630_30270	0.25	PTS system glucose-like IIA component	G	0.021
CD630_19220 (eutN)	0.25	Ethanolamine carboxysome structural protein	QC	0.016
CD630_20160	0.26	conserved hypothetical protein	-	0.034
23S_rRNA	0.27	23S ribosomal RNA	-	0.026
CD630_09360	0.28	putative phage endodeoxyribonuclease RusA-like	L	0.007
CD630_29341	0.29	putative phage protein	-	0.049
CD630_29470	0.30	putative phage protein	-	0.020
CD630_29320	0.30	putative phage protein	-	0.035
CD630_28780 (fhuD)	0.30	ABC-type transport system ferrichrome-specific extracellular solute-binding protein	P	0.004
CD630_14890 (metN)	0.32	ABC-type transport system methionine-specific ATP-binding protein	P	0.035
CD630_14900 (met I)	0.32	ABC-type transport system methionine-specific permease	P	0.034
CD630_16632	0.32	conserved hypothetical protein	-	0.018
CD630_17452	0.34	conserved hypothetical protein	R	0.026
CD630_21710	0.34	Fragment of putative sodium:dicarboxylate symporter	-	0.009
CD630_31360 (bglA7)	0.35	6-phospho-beta-glucosidase	G	0.001
CD630_26640 (murE)	0.35	UDP-N-acetylmuramyl-tripeptide synthetase	M	2.4E-04
CD630_28770 (fhuB)	0.35	ABC-type transport system ferrichrome-specific permease	P	1.5E-04
CD630_36010	0.35	D-alanyl-D-alanine carboxypeptidase M15 family	M	0.013
CD630_31000	0.36	putative C4-dicarboxylate anaerobic carrier Dcu family	S	0.046
CD630_18470	0.36	putative conjugative transposon protein Tn1549-like CTn5-Orf3	-	0.001
CD630_31150 (bglA4)	0.37	6-phospho-beta-glucosidase	G	0.003
CD630_13610	0.37	putative phage protein	-	0.002
CD630_32570	0.37	putative polysaccharide deacetylase	G	0.016
CD630_30340	0.38	Transcriptional regulator TrmB family	K	0.013
CD630_27491	0.38	Autoinducer prepeptide	-	2.5E-05
CD630_25150	0.38	putative L-aspartate-beta-decarboxylase	E	0.043
CD630_27640	0.38	putative hydrolase HAD superfamily IIB subfamily	R	0.003
CD630_26710	0.38	ABC-type transport system ATP-binding protein putative oligopeptide transport system	EP	0.028
CD630_33440	0.39	putative cell-division FtsK/SpoIIIE-family protein Tn916-like CTn6-Orf22	D	0.007
CD630_09340	0.39	putative phage protein	-	0.007
CD630_35370 (phnH)	0.39	putative phosphonate metabolism protein	P	0.007
CD630_31370 (bglF5)	0.39	PTS system beta-glucoside-specific IIAB component	G	0.022
CD630_30360	0.39	Transporter Major Facilitator Superfamily (MFS)	E	0.022
CD630_08450	0.40	putative nuclease	-	0.034
CD630_22010	0.40	Transporter Major Facilitator Superfamily (MFS)	G	0.045
CD630_25090	0.40	putative glycoside hydrolase family 4	G	0.016
CD630_23710 (nadB)	0.40	L-aspartate oxidase (Quinolinate synthetase B)	H	4.3E-04
CD630_23720 (nadA)	0.40	Quinolinate synthetase A	H	0.003
CD630_13640	0.40	putative phage XkdM-like protein	-	0.001
CD630_19180 (eutK)	0.40	Ethanolamine carboxysome strutural protein	QC	0.039
CD630_11540	0.40	Transcriptional regulator PadR family	K	0.025
CD630_03270 (cbiO)	0.40	ABC-type transport system cobalt-specific ATP-binding protein	P	0.003
CD630_05790	0.40	Transcriptional regulator TetR family	K	0.027
CD630_30990	0.40	putative amidohydrolase M20D family	R	0.031

CD630_03260 (cbiQ1)	0.41	ABC-type transport system cobalt-specific permease	P	0.002
CD630_18860	0.41	Transcriptional regulator PadR family	K	0.014
CD630_30720	0.41	conserved hypothetical protein	S	0.019
CD630_03140	0.41	putative membrane protein	S	0.018
CD630_26860	0.41	putative membrane protein	-	0.009
CD630_24620 (grpE)	0.41	HSP-70 cofactor	O	4.5E-04
CD630_29420	0.41	putative phage resolvase/integrase	-	1.4E-04
CD630_11261	0.42	Transcriptional regulator HTH-type	K	0.004
CD630_29400	0.42	putative phage protein	-	0.043
CD630_17540	0.42	ABC-type transport system multidrug-family permease	-	0.018
CD630_03240 (cbiM)	0.42	Cobalamin biosynthesis protein	P	0.009
CD630_23700 (nadC)	0.42	Nicotinate-nucleotide pyrophosphorylase	H	0.002
CD630_06140	0.43	conserved hypothetical protein	-	0.027
CD630_26700	0.43	ABC-type transport system ATP-binding protein putative oligopeptide transport system	E	0.025
CD630_01060 (cwlD)	0.43	Germination-specific N-acetylmuramoyl-L-alanineamidase Autolysin	M	0.009
CD630_26650	0.43	Transcriptional regulator AraC family	K	0.014
CD630_03901	0.43	conserved hypothetical protein	-	0.021
CD630_25160 (ansB)	0.44	L-asparaginase	EJ	0.034
CD630_29360	0.44	putative phage protein	-	0.007
CD630_05670	0.44	uncharacterised protein DegV family	S	0.033
CD630_01250	0.44	putative cell wall endopeptidase	M	0.046
CD630_04090	0.44	putative replication initiation protein Tn1549-like CTn2-Orf2	-	0.029
CD630_29440	0.44	putative phage essential recombination functionprotein	-	0.040
CD630_12700	0.45	Two-component sensor histidine kinase	T	0.001
CD630_30260	0.45	conserved hypothetical protein	TK	0.005
CD630_11710 (etfB4)	0.45	Electron transfer flavoprotein subunit alpha	C	0.008
CD630_29430	0.45	putative phage replication protein	L	0.010
CD630_09170	0.45	putative phage recombination protein Bet	-	6.1E-05
CD630_06240	0.45	putative transcriptional regulator activator	S	0.003
CD630_29710 (bioY)	0.45	Biotin synthase	R	0.012
CD630_01930 (groS)	0.46	chaperonin	O	0.003
CD630_20451	0.46	conserved hypothetical protein	-	1.9E-04
CD630_13450	0.46	Transcriptional regulator PadR family	K	0.002
CD630_26870	0.46	conserved hypothetical protein	-	0.007
CD630_29490	0.46	Transcriptional regulator Phage-type	-	0.005
CD630_05780	0.47	Transporter Major Facilitator Superfamily (MFS)	-	0.004
CD630_18270	0.47	Transcriptional regulator MarR family	K	0.033
CD630_30250	0.47	putative ferredoxin iron-sulphur domain-containing protein	C	0.007
CD630_32090	0.47	Transcriptional regulator PadR family	K	0.006
CD630_00470 (ispD)	0.48	2-C-methyl-D-erythritol 4-phosphatecytidylyltransferase	I	0.029
CD630_21430	0.48	Transcriptional regulator HTH-type	-	0.020
CD630_24610 (dnaK)	0.48	Chaperone protein dnaK (Heat shock protein 70)	O	0.009
CD630_25110	0.48	Transcription antiterminator PTS operon regulator	K	0.026
CD630_32100	0.48	conserved hypothetical protein	E	0.030
CD630_32620 (pstA)	0.48	ABC-type transport system phosphate-specific permease	P	0.045
CD630_25100	0.48	PTS system glucose-like IIBC component	G	0.043
CD630_27880	0.48	putative membrane protein GtrA family	S	0.004
CD630_13720	0.48	putative phage XkdT-like protein	-	2.2E-06
CD630_19210	0.48	putative ethanolamine utilization protein	-	0.017
CD630_10280	0.48	putative signaling protein	TK	0.030
CD630_21510	0.48	putative membrane protein DUF819 family	S	0.006
CD630_23300 (xpt)	0.49	Xanthine phosphoribosyltransferase (XPRTase)	F	0.036
CD630_15800 (hom2)	0.49	Homoserine dehydrogenase	E	0.010
CD630_24630 (hrcA)	0.49	Transcriptional regulator Heat-inducibler epressor	K	0.013
CD630_32220 (sdaB)	0.49	L-serine dehydratase	E	0.005
CD630_27500 (agrB)	0.49	Accessory gene regulator	OTK	0.001
CD630_32600 (phoU)	0.49	Phosphate uptake regulator	P	0.001
CD630_13460	0.50	conserved hypothetical protein DUF1700	S	0.002

CD630_26691	0.50	putative Na(+)/H(+) antiporter	P	0.009
CD630_29330	0.50	Hypothetical protein	J	0.010
CD630_03250 (cbiN)	0.50	ABC-type transport system cobalt-specific extracellular solute-binding protein	P	0.031

[a]Gene accession numbers are given for strain 630.
[b]Ratio of no LL-37/with LL-37 as determined by RNA sequencing analysis of 630Δ*erm* grown in BHIS alone or BHIS supplemented with 2 μg/ml LL-37 as described in Methods. Genes are included in this list if they had ≥ 2-fold increase or decrease in expression and a *P* value ≤ 0.05 by Student's two-tailed t-test.
[c]COG (classification of gene) designations are based on the 2014 COG database. Letter designations correspond to the categories listed in the table below.
[d]*P*-values determined by Student's two-tailed *t*-test.

A	RNA processing and modification
B	Chromatin Structure and dynamics
C	Energy production and conversion
D	Cell cycle control and mitosis
E	Amino Acid metabolism and transport
F	Nucleotide metabolism and transport
G	Carbohydrate metabolism and transport
H	Coenzyme metabolism
I	Lipid metabolism
J	Translation
K	Transcription
L	Replication and repair
M	Cell wall/membrane/envelop biogenesis
N	Cell motility
O	Post-translational modification, protein turnover, chaperone functions
P	Inorganic ion transport and metabolism
Q	Secondary Structure
T	Signal Transduction
U	Intracellular trafficking and secretion
Y	Nuclear structure
Z	Cytoskeleton
R	General Functional Prediction only
S	Function Unknown
-	Unassigned

Table S2. LL-37 MIC and MBC values for *clnR* and *clnAB* mutants

	MIC[a]	MBC[b]
630Δ*erm*	15	20
clnR	15	30
clnAB	15	20

[a] Minimum inhibitory concentration of LL-37 (μg/ml).
[b] Minimum bactericidal concentration of LL-37 (μg/ml).

Table S3. MIC values for *clnR* and *clnAB* mutants in various antimicrobials

	Van[a]	Amp	PmB	Nis
630Δ*erm*	1	4	500	360
clnR	1	4	500	360
clnAB	1	4	500	360

[a]Values shown are μg/ml. Van: vancomycin, Amp: ampicillin, PmB: polymyxin B, Nis: nisin.

Table S4. Genes differentially expressed in a *clnR* mutant in the presence/absence of LL-37

Gene[a]	*clnR*/ WT[b]	Product	COG[c]	*p*[d]
**ClnR activates (without LL-37)**				
CD630_16632	0.22	conserved hypothetical protein	-	0.003
CD630_23030	0.22	Fragment of putative phage transcriptional repressor	-	0.003
CD630_35370 (phnH)	0.22	putative phosphonate metabolism protein	P	9.0E-05
CD630_19280	0.22	putative membrane protein	D	9.0E-05
CD630_29341	0.23	putative phage protein	-	0.009
CD630_01631	0.24	conserved hypothetical protein	-	0.013
CD630_13700	0.25	putative phage XkdS-like protein	-	0.018
CD630_32770	0.26	PTS system mannose/fructose/sorbose IIC component	G	0.004
CD630_29330	0.32	Hypothetical protein	J	0.005
CD630_04090	0.33	putative replication initiation protein Tn1549-like CTn2-Orf2	-	0.032
CD630_00410	0.33	PTS system galactitol-specific IIA component	GT	2.7E-04
CD630_11980 (spoIIIAG)	0.33	Stage III sporulation protein AG	-	0.001
CD630_07400	0.35	putative pyridoxal phosphate-dependent aminotransferase	E	0.008
CD630_07420	0.37	putative ethanolamine transporter	E	0.003
CD630_36361	0.38	conserved hypothetical protein	-	0.015
CD630_29370	0.39	putative phage protein	-	0.011
CD630_01350	0.41	PTS system lactose/cellobiose-family IIA component	G	0.032
CD630_23260	0.41	PTS system fructose/mannitol family IIBc omponent	G	0.001
CD630_24170	0.42	PTS system Sorbitol-like IIB component	G	0.003
CD630_26870	0.43	conserved hypothetical protein	-	0.002
CD630_01981	0.43	conserved hypothetical protein	-	0.042
CD630_02850	0.44	PTS system mannose/fructose/sorbose IIB component	G	0.001
CD630_29490	0.44	Transcriptional regulator Phage-type	-	3.1E-04
CD630_13630	0.45	putative phage XkdK-like protein	-	0.016
CD630_29360	0.45	putative phage protein	-	0.009
CD630_16490	0.45	ABC-type transport system iron-family ATP-binding protein	P	0.022
CD630_21970	0.46	putative ferredoxin/flavodoxin oxidoreductase	C	0.017
CD630_12332	0.46	conserved hypothetical protein	-	0.034
CD630_26570	0.49	conserved hypothetical protein	-	0.006
CD630_04200	0.49	putative cell surface protein Tn1549-like CTn2-Orf15	R	0.006
CD630_21021	0.49	conserved hypothetical protein	-	0.006
CD630_04880 (orr)	0.50	putative small multidrug resistance SugE-like protein	P	0.041
**ClnR represses (without LL-37)**				
CD630_16180 (clnA)	57.26	ABC-type transport system multidrug-family ATP-binding protein	V	0.021
CD630_16190 (clnB)	53.69	ABC-type transport system multidrug-family permease	-	0.001
CD630_12400 (vanZ)	21.50	Teicoplanin resistance protein	V	0.017
CD630_33370	3.37	putative membrane protein Tn916-like CTn6-Orf14	B	0.006
CD630_07970	2.96	putative pyruvate carboxyltransferase	E	0.014
CD630_16310 (sodA)	2.70	spore coat protein-superoxide dismutase	P	4.3E-04
CD630_01820	2.47	conserved hypothetical protein	-	0.017
CD630_18840	2.43	conserved hypothetical protein	-	0.042
CD630_22180 (int2)	2.43	Fragment of putative integrase	-	0.042
CD630_03710	2.43	putative conjugative transposon protein Tn916-like CTn1-Orf16	-	0.042
CD630_15111	2.26	conserved hypothetical protein	-	0.037
CD630_16680	2.13	putative membrane protein	-	3.4E-06
CD630_27340	2.09	putative Na+/H+ antiporter NhaC family	C	0.001
CD630_12450	2.08	conserved hypothetical protein	-	0.032
**ClnR activates (with LL-37)**				
CD630_23540 (grdE)	0.06	Betaine reductase component B subunit	-	0.001
CD630_23520 (grdA)	0.07	Glycine reductase complex selenoprotein A (selenocysteine)	-	1.5E-04
CD630_02850	0.07	PTS system mannose/fructose/sorbose IIB component	G	0.002
CD630_02840	0.08	PTS system mannose/fructose/sorbose IIA component	G	0.005
CD630_23550 (trxA2)	0.09	Thioredoxin 2	O	0.005
CD630_23260	0.09	PTS system fructose/mannitol family IIB component	G	0.008
CD630_02880	0.10	PTS system mannose/fructose/sorbose IIC component	G	3.3E-04
CD630_16080	0.13	ABC-type transport system multidrug-family permease	-	0.003

CD630_02890	0.13	PTS system mannose/fructose/sorbose IID component	G	0.002
CD630_02860	0.13	PTS system mannose/fructose/sorbose IIA component	G	0.014
CD630_16060	0.13	Transcriptional regulator GntR family	K	0.001
CD630_16100	0.13	conserved hypothetical protein	-	0.001
CD630_16110	0.14	conserved hypothetical protein	-	0.003
CD630_02870	0.14	PTS system mannose/fructose/sorbose IIB component	G	0.002
CD630_23570 (grdX)	0.14	putative glycine reductase complex component	-	0.017
CD630_16090	0.15	conserved hypothetical protein	-	0.004
CD630_23560 (trxB3)	0.15	Thioredoxin reductase 3	O	0.018
CD630_23490 (grdC)	0.16	Glycine reductase complex component C	I	0.007
CD630_16070	0.16	ABC-type transport system multidrug-family ATP-binding protein	V	0.003
CD630_20751	0.18	conserved hypothetical protein	-	0.002
CD630_29290	0.18	putative phage protein	-	0.004
CD630_04200	0.20	putative cell surface protein Tn1549-like CTn2-Orf15	R	0.001
CD630_23240	0.21	putative sugar-phosphate dehydrogenase	ER	0.050
CD630_33740	0.21	putative conjugative transposon protein Tn916-like CTn7-Orf8	-	6.2E-05
CD630_23030	0.21	Fragment of putative phage transcriptional repressor	-	0.006
CD630_26000 (cstA)	0.22	Carbon starvation protein	T	1.4E-05
CD630_23810 (iorA)	0.22	Indole pyruvate ferredoxin/flavodoxin oxidoreductase	C	8.8E-05
CD630_04670	0.24	putative hydrolase HAD superfamily subfamily IIB	HR	0.001
CD630_23800 (iorB)	0.24	Indole pyruvate ferredoxin/flavodoxin oxidoreductase	C	4.0E-04
CD630_25990	0.24	putative transcriptional regulator	J	0.040
CD630_02900	0.25	conserved hypothetical protein	-	0.002
CD630_23820	0.26	putative pyridoxal phosphate-dependent transferase	E	0.002
CD630_29410	0.27	putative phage single-strand DNA-binding protein	L	0.037
CD630_16180 (clnA)	0.27	ABC-type transport system multidrug-family ATP-binding protein	V	0.005
CD630_33750 (mgtC)	0.27	Magnesium-transporting ATPase protein Tn916-like CTn7-Orf10	S	0.002
CD630_16190 (clnB)	0.27	ABC-type transport system multidrug-family permease	-	0.011
CD630_33730 (mgtA)	0.27	Magnesium-transporting ATPase P-type Tn916-like CTn7-Orf7	P	0.001
CD630_00330	0.28	putative glycoside hydrolase	G	0.048
CD630_07400	0.28	putative pyridoxal phosphate-dependent aminotransferase	E	0.045
CD630_05490	0.28	conserved hypothetical protein	-	0.001
CD630_33741	0.29	putative conjugative transposon protein Tn916-like CTn7-Orf9	-	0.002
CD630_28130 (garR)	0.30	Tartronate semialdehyde reductase	I	0.042
CD630_29370	0.31	putative phage protein	-	0.001
CD630_00220 (fusA1)	0.31	Elongation factor G	J	0.013
CD630_18570	0.31	putative cell wall hydrolase Tn1549-like CTn5-Orf13	M	0.038
CD630_18880 (rsiU)	0.32	Extracytoplasmic function (ECF) anti-sigma factor	-	0.004
CD630_05000	0.32	putative antirestriction protein Tn5397 CTn3-Orf18	V	0.006
CD630_17970	0.34	Coenzyme A disulfide reductase	R	0.037
CD630_01350	0.34	PTS system lactose/cellobiose-family IIA component	G	0.048
CD630_04960	0.34	putative conjugative transposon protein DUF961 family Tn5397 CTn3-Orf23	-	0.007
CD630_23480 (grdD)	0.34	Glycine reductase complex component C	I	0.015
CD630_04980	0.34	putative cell-division FtsK/SpoIIIE-family protein Tn5397 CTn3-Orf21	D	0.001
CD630_17000 (ribD)	0.35	Riboflavin biosynthesis protein	H	0.014
CD630_28620	0.36	putative peptidase M19 family	E	0.016
CD630_18560	0.36	putative hydrolase Tn1549-like CTn5-Orf12	U	0.002
CD630_15970	0.36	conserved hypothetical protein	-	3.0E-04
CD630_28240	0.36	Fragment of putative membrane protein	-	0.020
CD630_33721	0.36	putative conjugative transposon protein Tn916-like CTn7-Orf5	-	0.004
CD630_04970	0.36	putative conjugative transposon protein DUF961 family Tn5397 CTn3-Orf22	-	0.008
CD630_05103	0.37	putative conjugative transposon proteinTn5397%2CCTn3-Orf4	-	0.013
CD630_04990	0.38	putative replication initiation factor Tn5397 CTn3-Orf20	L	0.001
CD630_22820	0.39	PTS system fructose/mannitol family IIA component	GT	0.024
CD630_04991	0.39	putative conjugative transposon protein Tn5397 CTn3-Orf19	-	0.005
CD630_02910	0.40	putative peptidase M20A family	E	0.009
CD630_16980 (ribBA)	0.40	GTP cyclohydrolase-2 Riboflavin biosynthesis protein	H	0.012
CD630_05100	0.40	putative RNA polymerase sigma factor Tn5397 CTn3-Orf7	K	0.001
CD630_17960	0.40	putative nitrite and sulfite reductase subunit	C	0.044

CD630_05102	0.40	Fragment of putative conjugative transposon protein Tn5397 CTn3-Orf5	-	0.002
CD630_05060	0.41	Reverse transcriptase/maturase/endonuclease Group II intron	V	0.001
CD630_33722	0.41	putative conjugative transposon protein Tn916-like CTn7-Orf6	-	0.002
CD630_23790 (buk2)	0.41	Butyrate kinase	C	2.5E-04
CD630_18870 (csfU)	0.41	Extracytoplasmic function (ECF) sigma factor	K	0.001
CD630_28280	0.41	putative pyridoxal phosphate-dependent transferase	E	0.029
CD630_05101	0.41	putative conjugative transposon protein Tn5397 CTn3-Orf8	-	1.6E-04
CD630_07390	0.42	putative exported protein	-	1.0E-04
CD630_15431	0.43	conserved hypothetical protein	-	0.015
CD630_20750 (pbuX)	0.43	putative xanthine permease	F	0.001
CD630_28110	0.43	conserved hypothetical protein	S	0.010
CD630_08650	0.44	putative ADP-ribose binding protein	R	0.013
CD630_06790	0.45	conserved hypothetical protein	R	0.018
CD630_04890	0.45	putative phosphoribosylaminoimidazole-succinocarboxamide synthetase	F	0.001
CD630_23590	0.46	putative hydrolase HAD superfamily subfamily IIB	R	2.9E-04
CD630_28590	0.46	putative D-aminoacylase	Q	0.018
CD630_18890	0.47	ABC-type transport system multidrug-family ATP-binding protein	V	0.004
CD630_06770 (csfT)	0.47	Extracytoplasmic function (ECF) sigma factor	K	0.001
CD630_21600	0.47	putative membrane protein	V	0.019
CD630_16220	0.47	peptidase propeptide and ypeb domain protein	S	0.034
CD630_24020	0.47	putative cell wall hydrolase phosphatase-associated protein	M	0.008
CD630_18900	0.47	ABC-type transport system multidrug-family permease	O	0.007
CD630_04400 (cwp27)	0.47	putative cell wall binding protein	-	8.9E-05
CD630_29490	0.48	Transcriptional regulator Phage-type	-	0.004
CD630_27080 (aroE2)	0.48	Shikimate dehydrogenase 2	E	8.6E-05
CD630_09370	0.48	putative phage anti-repressor	K	0.012
CD630_25670	0.49	PTS system mannose-specfic IIB component	G	0.009
CD630_05020	0.49	putative ATPase Tn5397 CTn3-Orf16	L	0.002
CD630_29300	0.49	putative phage anti-repressor protein	K	0.012
CD630_27840 (cwp6)	0.49	putative N-acetylmuramoyl-L-alanineamidase autolysin	M	0.002
CD630_01420	0.49	putative RNA-binding protein	J	0.028
CD630_22490	0.50	putative ATPase	R	0.001
CD630_21910	0.50	putative phosphoesterase	ER	0.008
CD630_23090	0.50	conserved hypothetical protein	-	0.039
**ClnR represses (with LL-37)**				
CD630_26140	7.62	uncharacterised protein DegV family	S	0.017
CD630_06680	4.19	Two-component response regulator	K	0.014
CD630_06690	4.01	Two-component sensor histidine kinase	T	0.011
CD630_27491 (agrD)	3.46	Autoinducer prepeptide	-	0.002
CD630_11920	3.10	Stage III sporulation protein AA	S	0.039
CD630_24291	3.10	putative 4Fe-4S ferredoxin iron-sulfur binding domain protein	C	0.039
CD630_28370	2.74	putative membrane protein	S	0.046
CD630_04370	2.74	Fragment of integrase Tn1549-like CTn2	-	0.046
CD630_09780	2.52	Transcriptional regulator beta-lactams repressor Phage-type	-	0.011
CD630_22290	2.49	putative membrane protein	S	0.049
CD630_10950	2.48	ABC-type transport system multidrug-family permease Tn1549-like CTn4-Orf30	-	0.030
CD630_25150	2.45	putative L-aspartate-beta-decarboxylase	E	0.039
CD630_04640	2.45	putative beta-lactamase-like hydrolase	R	0.018
CD630_26640 (murE)	2.42	UDP-N-acetylmuramyl-tripeptide synthetase	M	0.009
CD630_09950 (serA)	2.40	putative D-3-phosphoglycerate dehydrogenase	HE	0.019
CD630_09960	2.34	conserved hypothetical protein	S	0.015
CD630_21510	2.34	putative membrane protein DUF819 family	S	0.007
CD630_33360	2.32	putative cell wall hydrolase Tn916-like CTn6-Orf13	M	5.0E-04
CD630_01260 (spoIIID)	2.29	Stage III sporulation protein D	-	0.002
CD630_30730	2.24	putative membrane protein	-	0.008
CD630_10970	2.22	ABC-type transport system multidrug-family ATP-binding protein Tn1549-like CTn4-Orf28	V	0.008
CD630_25290	2.18	conserved hypothetical protein	-	0.021
CD630_21710	2.18	Fragment of putative sodium:dicarboxylate symporter	-	0.036

CD630_08220	2.11	ABC-type transport system multidrug-family ATP-binding protein	V	0.016
CD630_26700	2.10	ABC-type transport system ATP-binding protein putative oligopeptide transport system	E	0.031
CD630_25940 (uraA)	2.10	ABC-type transport system uracil-specific permease	F	4.5E-04
CD630_08230	2.05	ABC-type transport system multidrug-family permease	-	0.042
CD630_30230	2.03	conserved hypothetical protein	-	0.034
CD630_23750	2.03	uncharacterised protein	-	0.022

[a]Gene accession numbers are given for strain 630. Gene *CD630_20072 (ermB*) is excluded from these tables because it is an artifact of the insertional disruption used to generate the *clnR* mutant.
[b]Ratio of expression in *clnR* mutant/630Δ*erm*, as determined by RNA sequencing analysis of 630Δ*erm* and *clnR* grown in BHIS alone or BHIS supplemented with 2 μg/ml LL-37 as described in Methods. Fold-change represents the ratio of expression in *clnR*/630Δ*erm* in the condition indicated (with or without LL-37). Genes are included in this list if they had ≥ 2-fold increase or decrease in expression and a *p* value ≤ 0.05 by Student's two-tailed *t*-test.
[c]COG designations are based on the 2014 COG database. Letter designations correspond to the categories listed in the table associated with Table S1.
[d]*p* values determined by Student's two-tailed *t*-test.

Table S5. Genes regulated by both ClnR and LL-37

Gene[a]	Fold change in LL-37[b]	*clnR/*WT[c]	*clnR*/WT (+ LL-37)[d]	Product
CD630_01631	0.22	0.24	1.63	spore coat protein-superoxide dismutase
CD630_02910	3.25	1.32	0.40	putative peptidase M20A family
CD630_04090	0.44	0.33	0.87	putative replication initiation protein Tn1549-like CTn2-Orf2
CD630_04670	3.43	1.44	0.24	putative hydrolase HAD superfamily subfamily IIB
CD630_04890	2.26	1.04	0.45	putative phosphoribosylaminoimidazole-succinocaroxamide synthetase
CD630_04960	4.13	1.19	0.34	putative conjugative transposon protein DUF961 family Tn5397 CTn3-Orf23
CD630_04970	3.86	0.93	0.36	putative conjugative transposon protein DUF961 family Tn5397 CTn3-Orf22
CD630_04980	4.13	0.93	0.34	putative cell-division FtsK/SpoIIIE-family protein Tn5397 CTn3-Orf21
CD630_05000	3.61	1.08	0.32	putative antirestriction protein Tn5397 CTn3-Orf18
CD630_05060	3.54	0.97	0.41	Reverse transcriptase/maturase/endonuclease Group II intron
CD630_05100	3.07	0.79	0.40	putative RNA polymerase sigma factor Tn5397 CTn3-Orf7
CD630_05101	3.31	1.05	0.41	putative conjugative transposon protein Tn5397 CTn3-Orf8
CD630_05102	3.35	0.89	0.40	Fragment of putative conjugative transposon protein Tn5397 CTn3-Orf5
CD630_05103	3.27	1.06	0.37	putative conjugative transposon protein Tn5397 CTn3-Orf4
CD630_05490	4.74	1.15	0.28	conserved hypothetical protein
CD630_05500	5.52	0.81	0.53	putative membrane protein
CD630_09950 (serA)	0.23	1.14	2.40	putative D-3-phosphoglycerate dehydrogenase
CD630_09960	0.24	1.19	2.34	conserved hypothetical protein
CD630_12400 (vanZ)	28.43	21.50	0.74	Teicoplanin resistance protein
CD630_16070	30.25	1.02	0.16	ABC-type transport system multidrug-family ATP-binding protein
CD630_16090	30.62	1.15	0.15	conserved hypothetical protein
CD630_16100	64.45	2.17	0.13	conserved hypothetical protein
CD630_16110	34.77	1.22	0.14	conserved hypothetical protein
CD630_16180 (clnA)	202.99	57.26	0.27	ABC-type transport system multidrug-family ATP-binding protein
CD630_16190 (clnB)	192.16	53.69	0.27	ABC-type transport system multidrug-family permease
CD630_16310 (sodA)	2.59	2.70	0.88	spore coat protein-superoxide dismutase
CD630_16632	0.32	0.22	3.61	conserved hypothetical protein
CD630_17000 (ribD)	3.65	1.11	0.35	Riboflavin biosynthesis protein
CD630_18870 (csfU)	5.34	1.24	0.41	Extracytoplasmic function (ECF) sigma factor
CD630_18880 (rsiU)	3.69	0.95	0.32	Extracytoplasmic function (ECF) anti-sigma factor
CD630_18890	4.54	1.40	0.47	ABC-type transport system multidrug-family ATP-binding protein
CD630_18900	3.60	1.17	0.47	ABC-type transport system multidrug-family permease
CD630_20140 (ilvD)	2.90	1.02	0.53	Dihydroxy-acid dehydratase
CD630_20751	2.21	0.51	0.18	conserved hypothetical protein
CD630_21510	0.48	1.11	2.34	putative membrane protein DUF819 family
CD630_21710	0.34	1.05	2.18	Fragment of putative sodium:dicarboxylate symporter
CD630_22290	0.51	1.02	2.49	putative membrane protein
CD630_23090	2.29	1.03	0.50	conserved hypothetical protein
CD630_23790 (buk2)	3.13	0.81	0.41	Butyrate kinase
CD630_23800 (iorB)	10.81	0.64	0.24	Indole pyruvate ferredoxin/flavodoxin oxidoreductase
CD630_23810 (iorA)	13.66	0.62	0.22	Indole pyruvate ferredoxin/flavodoxin oxidoreductase
CD630_25150	0.38	1.02	2.45	putative L-aspartate-beta-decarboxylase

CD630_26140	0.12	1.18	7.62	uncharacterised protein DegV family
CD630_26640 (murE)	0.35	1.08	2.42	UDP-N-acetylmuramyl-tripeptide synthetase
CD630_26700	0.43	0.91	2.10	ABC-type transport system ATP-binding protein putative oligopeptide transport system
CD630_26870	0.46	0.43	0.79	conserved hypothetical protein
CD630_27491	0.38	0.92	3.46	Autoinducer prepeptide
CD630_27840 (cwp6)	2.30	0.97	0.49	putative N-acetylmuramoyl-L-alanineamidase autolysin
CD630_29330	0.50	0.32	1.45	Hypothetical protein
CD630_29341	0.29	0.23	1.34	putative phage protein
CD630_29360	0.44	0.45	0.57	putative phage protein
CD630_29490	0.46	0.44	0.48	Transcriptional regulator Phage-type
CD630_33730 (mgtA)	3.39	0.78	0.27	Magnesium-transporting ATPase P-type Tn916-like CTn7-Orf7
CD630_33740	3.92	0.76	0.21	putative conjugative transposon protein Tn916-like CTn7-Orf8
CD630_33750 (mgtC)	2.96	0.57	0.27	Magnesium-transporting ATPase protein Tn916-like CTn7-Orf10
CD630_35370 (phnH)	0.39	0.22	1.96	putative phosphonate metabolism protein

[a]Gene accession numbers are given for strain 630. Genes are included in this list if they had ≥ 2-fold increase or decrease in expression and a *p* value ≤ 0.05 by Student's two-tailed *t*-test in both 630Δ*erm* in LL-37 and the *clnR* mutant (either with or without LL-37).
[b]Ratio of no LL-37/with LL-37 as determined by RNA sequencing analysis of 630Δ*erm* grown in BHIS alone or BHIS supplemented with 2 μg/ml LL-37 as described in Methods.
[c]Ratio of expression in *clnR* mutant/expression in 630Δ*erm* as determined by RNA sequencing analysis of strains grown in BHIS alone as described in Methods.
[d]Ratio of expression in *clnR* mutant/expression in 630Δ*erm* as determined by RNA sequencing analysis of strains grown in BHIS supplemented with 2 μg/ml LL-37 as described in Methods.

Table S6. Relative expression of selected RNA-seq transcripts in *clnR* and *clnAB* mutants

Gene	BHIS					+ LL-37 (2 µg/ml)				
	630Δ*erm*	*clnR*	*clnAB*	*clnR* Tn::*clnRAB*	*clnAB* Tn::*clnRAB*	630Δ*erm*	*clnR*	*clnAB*	*clnR* Tn::*clnRAB*	*clnAB* Tn::*clnRAB*
cdd4 (CD630_06670)	1.0 ± 0.0[a]	0.9 ± 0.1	1.1 ± 0.2	0.9 ± 0.2	1.0 ± 0.2	7.1*± 2.4	**915.0***± 30.5	**1672.6*** ± 357	57.2*± 16.1	314.8*± 98.0
vanZ1 (CD630_12400)	1.0 ± 0.0	**25.2** ± 5.5	0.8 ± 0.1	0.8 ± 0.1	0.6 ± 0.0	36.9*± 6.0	**13.4** ± 5.8	**0.9** ± 0.2	**81.5*** ± 7.5	**0.6** ± 0.1
cstA (CD630_26000)	1.0 ± 0.0	0.9 ± 0.2	1.2 ± 0.1	0.6 ± 0.1	0.8 ± 0.2	6.7*± 1.5	2.3 ± 0.5	2.9 ± 1.2	36.3 ± 21.3	1.3 ± 0.2
sigU (csfU; CD630_18870)	1.0 ± 0.0	0.5 ± 0.2	1.3 ± 0.3	0.6 ± 0.1	0.7 ± 0.1	10.5 ± 6.8	2.0 ± 0.6	3.1 ± 1.4	**25.6***± 1.9	0.8 ± 0.1
sigT (csfT; CD630_06770)	1.0 ± 0.0	0.9 ± 0.1	1.0 ± 0.2	1.1 ± 0.1	1.1 ± 0.1	3.6*± 0.1	2.3 ± 1.1	1.2 ± 0.1	**25.3***± 1.7	1.2 ± 0.2
grdA (CD630_23520)	1.0 ± 0.0	0.9 ± 0.1	1.0 ± 0.0	0.9 ± 0.1	1.3 ± 0.2	16.5 ± 10.5	1.4 ± 0.1	1.9* ± 0.1	**1649.2** ± 814.6	1.6 ± 0.2
mtlA (CD630_23340)	1.0 ± 0.0	0.9 ± 0.4	3.3 ± 2.1	0.6 ± 0.2	0.5 ± 0.1	1.0 ± 0.3	1.2 ± 0.7	5.5 ± 4.3	0.6 ± 0.2	0.7 ± 0.4
iorA (CD630_23810)	1.0 ± 0.0	**0.5** ± 0.1	0.7 ± 0.1	0.7 ± 0.0	1.1 ± 0.2	16.8 ± 8.4	2.1*± 0.4	20.5 ± 7.3	**112.4***± 19.9	2.8*± 0.3
CD630_02840	1.0 ± 0.0	1.2 ± 0.1	1.1 ± 0.1	1.1 ± 0.2	1.3 ± 0.2	29.3 ± 18.8	2.2 ± 0.5	2.1* ± 0.2	602.6 ± 455.6	2.8*± 0.1

[a]Relative expression levels were determined by qRT-PCR and are normalized to 630Δ*erm* in BHIS as described in methods. Values shown are the mean of at least 3 biological replicates ± standard error of the mean. Data were analyzed by one-way ANOVA and Dunnett’ s multiple comparisons test for comparisons to 630Δ*erm* in the same condition or by Student’s *t* test with Holm-Sidak correction for comparisons of the same strain with/without LL-37. Bolded values indicate an adjusted *p* value < 0.05 comparing to 630Δ*erm* in the same condition; * indicates an adjusted *p* value < 0.05 comparing the same strain with/without LL-37.

Table S7. Maximum doubling times for 630Δ*erm*, *clnR, clnAB, clnR* Tn::*clnRAB,* and *clnAB* Tn::*clnRAB* in minimal media supplemented with metabolites, with or without LL-37

MM+[a]	strain	No LL-37 Doubling time (min)[b]	LL-37[c] Doubling time (min)[b]
--	630Δ*erm*	73.2 ± 3.4	82.2 ± 5.1
	clnR	76.3 ± 2.6	74.1 ± 4.8
	clnAB	73.5 ± 2.3	77.1 ± 5.3
	clnR Tn::*clnRAB*	71.6 ± 2.5	§84.8 ± 4.5
	clnAB Tn::*clnRAB*	71.7 ± 2.0	76.4 ± 2.6
Glucose	630Δ*erm*	66.2 ± 3.7	67.7 ± 4.9
	clnR	68.4 ± 3.5	74.0 ± 1.7
	clnAB	*61.4 ± 3.3	62.1 ± 2.2
	clnR Tn::*clnRAB*	64.6 ± 3.7	76.4 ± 5.4
	clnAB Tn::*clnRAB*	63.6 ± 2.5	70.8 ± 4.8
Fructose	630Δ*erm*	64.8 ± 4.1	70.2 ± 3.9
	clnR	70.6 ± 1.7	72.4 ± 0.9
	clnAB	63.9 ± 3.5	65.1 ± 2.0
	clnR Tn::*clnRAB*	67.8 ± 2.5	80.4 ± 7.4
	clnAB Tn::*clnRAB*	67.2 ± 2.6	69.8 ± 2.5
Mannose	630Δ*erm*	71.0 ± 5.2	70.3 ± 5.5
	clnR	70.5 ± 2.9	78.4 ± 4.9
	clnAB	65.6 ± 3.7	66.8 ± 4.5
	clnR Tn::*clnRAB*	72.4 ± 4.6	80.4 ± 8.3
	clnAB Tn::*clnRAB*	68.9 ± 3.4	72.6 ± 3.1
Mannitol	630Δ*erm*	60.0 ± 2.1	69.9 ± 3.4
	clnR	63.7 ± 0.4	67.3 ± 1.5
	clnAB	60.1 ± 3.2	58.3 ± 5.5
	clnR Tn::*clnRAB*	64.5 ± 2.6	75.6 ± 5.3
	clnAB Tn::*clnRAB*	*62.4 ± 0.7	66.4 ± 1.8
NAG	630Δ*erm*	66.6 ± 2.7	§91.5 ± 5.7
	clnR	81.8 ± 1.5	86.6 ± 2.5
	clnAB	70.9 ± 0.8	§80.3 ± 1.0
	clnR Tn::*clnRAB*	73.6 ± 3.1	111.1 ± 16.5
	clnAB Tn::*clnRAB*	*72.4 ± 1.4	§83.6 ± 1.7
EA	630Δ*erm*	78.4 ± 4.0	90.7 ± 7.9
	clnR	*89.9 ± 3.2	84.9 ± 4.6
	clnAB	87.7 ± 7.0	79.6 ± 9.0
	clnR Tn::*clnRAB*	86.2 ± 4.7	93.4 ± 12.2
	clnAB Tn::*clnRAB*	85.9 ± 7.2	84.0 ± 6.7

[a]Strains were grown in MM with additional nutrients as listed: 30mM glucose, 30 mM fructose, 30 mM mannose, 30 mM N-acetylglucosamine (NAG), 30 mM mannitol, 30 mM ethanolamine (EA)
[b]Doubling time was calculated during the 2 hour period of maximal growth after the conclusion of peptide-fueled growth, where $t_d = \ln(2)/\mu$, where $\mu = (\ln(OD_{t2}) - \ln(OD_{t1}))/(t_2 - t_1)$[1, 2]. Values shown are the average of at least three independent experiments ± standard deviation.
[c]LL-37, 0.5 µg/ml
§To compare +/- LL-37 for each strain/substrate (across rows), data were analyzed by Student's *t* test (**§**indicates $p < 0.05$).
To compare the growth of strains in each condition (within boxes), data were analyzed by one-way ANOVA with Dunnett's test for multiple comparisons (indicates adjusted $p < 0.05$)

Table S8. Expression of toxin regulation-associated genes

Gene	Fold-change in expression LL-37[a, b]
sigD	0.95 ± 0.08
tcdR	1.47 ± 0.44
ilvC[c]	**2.21** ± 0.20
CD0341[d]	2.54 ± 0.59

[a]Fold-change determined by qRT-PCR and normalized to 630Δ*erm* grown in BHIS alone. Concentration of LL-37 = 2 μg/ml. Values are the mean of three replicates ± standard error of the mean.
[b]Bolded values indicate significant difference (*p* value < 0.05) from 630Δ*erm* grown in BHIS without LL-37 and analyzed by Student's two-tailed *t*-test.
[c]*ilvC* indicates CodY activity.
[d]*CD0341* indicates CcpA activity.

Table S9. Plasmids and Strains

Plasmid or Strain		Relevant genotype or features	Source, construction or reference
**Strains**			
E. coli			
	HB101	F$^-$ *mcrB mrr hsdS20*(r_B^- m_B^-) *recA13 leuB6 ara-14 proA2 lacY1 galK2 xyl-5 mtl-1 rpsL20*	B. Dupuy
	MC101	HB101 pRK24	B. Dupuy
	MC135	HB101 pRK24 pMC123	(McBride and Sonenshein, 2011)
	MC881	HB101 pRK24 pMC616	
	MC932	HB101 pRK24 pMC645	
	MC1122	HB101 pRK24 pMC723	
B. subtilis	BS49	*Tn*916	
	MC951	BS49 *Tn*916::*CD1617-1619*	
C. difficile			
	630	Clinical isolate	[3]
	630Δ*erm*	Erm^S derivative of strain 630	N. Minton [4]
	R20291	Clinical isolate	[5]
	MC324	630Δ*erm* pMC123	(Edwards et al., 2014)
	MC885	630Δ*erm CD1617::ermB*	
	MC935	630Δ*erm CD1618::ermB*	
	MC950	MC885 *Tn*916::*CD1617-1619*	
	MC953	MC935 *Tn*916::*CD1617-1619*	
	MC1123	MC885 pMC123	
	MC1131	MC885 pMC723	
**Plasmids**	pRK24	Tra$^+$, Mob$^+$; *bla, tet*	[6]
	pCR2.1	*bla, kan*	Invitrogen
	pUC19	Cloning vector; *bla*	[7]
	pCE240	*C. difficile* TargeTron→ construct based on pJIR750ai (group II intron, *ermB::*RAM, *ltrA*); *catP*	C. Ellermeier; [8]
	pSMB47	Tn*916* integrational vector; CmR, ErmR	[9]
	pMC123	*E. coli-C. difficile* shuttle vector; *bla, catP*	
	pMC577	pCR2.1 with *clnR*-targeted intron	
	pMC602	pCE240 with *clnR*-targeted intron	
	pMC616	pMC123 with *clnR*-targeted intron (~nt 127, *ermB::*RAM *ltrA catP*	
	pMC643	pCE240 with *clnA*-targeted intron	
	pMC645	pMC123 with *clnA*-targeted intron (~nt 217, *ermB*::RAM *ltrA catP*	
	pMC649	pSMB47 Tn*916*::*CD1617-1619*	
	pMC723	pMC123 with P_{clnR}::His-*clnRAB*	

Table S10. Oligonucleotides

Primer	Sequence[a]	Purpose, source, or reference[b]
oMC44	CTAGCTGCTCCTATGTCTCACATC	*rpoC* (*CD0067*) qPCR [10]
oMC45	CCAGTCTCTCCTGGATCAACTA	*rpoC* (*CD0067*) qPCR [10]
oMC112	GGCAAATGTAAGATTTCGTACTCA	*tcdB* (*CD0660*) qPCR [11]
oMC113	TCGACTACAGTATTCTCTGAC	*tcdB* (*CD0660*) qPCR [11]
oMC152	GTTATGGAAGTCAAGGACATGCAC	*ilvC (CD1565*) qPCR [12]
oMC153	GCTTCTGCTACACTCTTAACTTCA	*ilvC (CD1565*) qPCR [12]
oMC178	CTTGAGTTAAATCTTGTGCAGTCA	*csfU (CD1887*) qPCR
oMC179	GGTGATAATAGTGAATGATGCTCGG	*csfU (CD1887*) qPCR
oMC189	TGCCTCTTGTAAAGAGTATAGCA	*sigD (CD0266*) qPCR [11]
oMC190	GCATCAATCAATCCAATGACTCCAC	*sigD (CD0266*) qPCR [11]
oMC242	TCCACAAGGAGCTGTATATGGT	*cdd4 (CD0667)* qPCR
oMC243	GTGGGTTTAGCAAGTCCAAGAA	*cdd4 (CD0667)* qPCR
oMC547	TGGATAGGTGGAGAAGTCAGT	*tcdA (CD0663*) qPCR [11]
oMC548	GCTGTAATGCTTCAGTGGTAGA	*tcdA (CD0663*) qPCR [11]
oMC683	GTATCTGACAACATCAATTGCCTAAA	*CD0341* qPCR [11]
oMC684	TCAGCTTGAGATTCAATTTCTTCATT	*CD0341* qPCR [11]
oMC815	TGGATTCTCTTAAGGAAGAACAATACTTTA	*sigT* (*CD0677*) qPCR [13]
oMC816	CCTTAACTTCATCTACTGAATAACCTTCA	*sigT* (*CD0677*) qPCR [13]
oMC1249	GTCGAGGATCCGATGACAAGTTATTGGAATACACAG	P*spo0A* amplification
oMC1290	GAATGGGAACTTGATAATAACAAACC	check *CD1617-1618* co-transcription; *clnR* (*CD1617*) qPCR
oMC1291	AAGTTCTGTTAGAGCCTTTTGC	check *CD1616-1617* co-transcription; *clnR* (*CD1617*) qPCR
oMC1292	AGGTGTAAACAAGAGTTATGGAAC	check *CD1618-1619* co-transcription; *clnA* (*CD1618*) qPCR
oMC1293	TCTATGGATGGTTTCATTCCATTTATC	check *CD1617-1618* co-transcription; *clnA* (*CD1618*) qPCR
oMC1294	AAGCAAGTGGAAGAATATTTATACCG	*clnB* (*CD1619*) qPCR
oMC1295	ACATTAAATAACCTTCATCCCCC	*clnB* (*CD1619*) qPCR
oMC1297	CACTGCAGTTTTATCCATTTTATAATTC	screening for Targetron insertion in *clnR*
oMC1310	AAAAGCTTTTGCAACCCACGTCGATCGTGAACCGCATCTTCTGGTGCGCCCAGATAGGGT	*clnR (CD1617)* intron retargeting
oMC1311	CAGATTGTACAAATGTGGTGATAACAGATAAGTCCTTCTGCTTAACTTACCTTTCTTTGT	*clnR (CD1617)* intron retargeting
oMC1312	CGCAAGTTTCTAATTTCGGTTTGCGGTCGATAGAGGAAAGTGTCT	*clnR (CD1617)* intron retargeting
oMC1319	AAAAGCTTTTGCAACCCACGTCGATCGTGAAAAAATAGTTTCAGTGCGCCCAGATAGGGT	*clnA (CD1618)* intron retargeting
oMC1320	CAGATTGTACAAATGTGGTGATAACAGATAAGTCGTTTCATATAACTTACCTTTCTTTGT	*clnA (CD1618)* intron retargeting
oMC1321	CGCAAGTTTCTAATTTCGGTTATTTTTCGATAGAGGAAAGTGTCT	*clnA (CD1618)* intron retargeting
oMC1383	GTAGAAGGAGCAGAGGTTGTTT	*grdA (CD2352*) qPCR
oMC1384	TCAGCAGCATCTTTAACTCTGT	*grdA (CD2352*) qPCR
oMC1393	TGAAACCATGAATCTTAGAAGCATAAAC	*vanZ (CD1240)* qPCR
oMC1394	CACATATATCCCAAATGGTACAAATATAGC	*vanZ (CD1240)* qPCR
oMC1410	GTGGGATCCGCTAAAACTTATTACAG	*clnA (CD1618*) cloning
oMC1416	GTGGGATCCAGAAGAACAGTTTAA	P*clnR* cloning
oMC1427	GTTTGGAAAGCCAATGCCAA	check *CD1616-1617* co-transcription
oMC1467	TAGCAGAAGATGCGGAAGTTAAT	*clnR (CD1617*) qPCR
oMC1473	GTTACAAATCTTCCTTTAGTTCTCTGAC	*clnR (CD1617*) qPCR
oMC1476	GCGCATGCATTACTCAAAAGATAGCT	*clnRAB* cloning
oMC1483	CTGGGTCAACACCACCTATAG	verify *clnA (CD1618)* disruption
oMC1493	GTTAGAAGAGCAAATGAGATGATTAAAAGC	*clnA (CD1618*) qPCR
oMC1614	GTGTACTCCACCAGCAAAGA	*cstA* (*CD2600*) qPCR
oMC1615	GCAGGGTTAGGTCCGATATTT	*cstA* (*CD2600*) qPCR
oMC1684	GCGGAATTCCTAAAAGTAATTGACATATACTTTG	P*clnR* cloning
oMC1689	GTGGGATCCGGCGCCATGCATCACCATCACCATCACATGGAATGGGAACTTGATAATAAC	*clnR* cloning with His-tag

oMC1690	GTGGGCGCCGTTCATGCCTCCTTATTA	P*clnR* cloning
oMC1691	FAM-CTAAAAAGTAATTGACATATACTTTG	P*clnR* amplification with fluorescein
oMC1692	FAM-CATGCCTCCTTATTATATTATTG	P*clnR* amplification with fluorescein
oMC1700	FAM-AGTTTGTGCAGTTTCTGAA	P*iorA* amplification with fluorescein
oMC1701	FAM-ACTACAATTATTAAATTCATAGATG	P*iorA* amplification with fluorescein
oMC1702	FAM-CTCCAAAATACTACATAAATAA	P*mtlA* amplification with fluorescein
oMC1703	FAM-TATATCGATATGATTCCCTTTTG	P*mtlA* amplification with fluorescein
oMC1704	FAM-CAAATTAAAATAAAGCAATTTATA	P*CD1606* amplification with fluorescein
oMC1705	FAM-CTAGTGTATTAATACGATAGTAC	P*CD1606* amplification with fluorescein
oMC1706	FAM-TTGTTTAAGTATTAATTATGAGT	P*csfU* amplification with fluorescein
oMC1707	FAM-CGTCATTATATATAACGATTTATAC	P*csfU* amplification with fluorescein
oMC1710	FAM-ATGTTTCATCCCCTTTTTTAATC	P*cdd4/CD0668* amplification with fluorescein
oMC1711	FAM-CACCCTCCTTTAGTATACC	P*cdd4/CD0668* amplification with fluorescein
oMC1712	FAM-CAATATTAATTTATTTTTAAAAAATAG	P*tcdA* amplification with fluorescein
oMC1713	FAM-AGTATTATTATTTTTGATAATAAATC	P*tcdA* amplification with fluorescein
oMC1714	FAM-CTTGTAATAAAATAAAGATTTAAGTG	P*grdE* amplification with fluorescein
oMC1715	FAM-CACCTCCTGTTATTTAATTTG	P*grdE* amplification with fluorescein
oMC1716	CACCAATAATTTTATTATTTTGTATTATTG	P*spo0A* amplification
oMC1735	AGAAAGATATGAAATACTACAATAGC	P*vanZ* amplification with fluorescein
oMC1736	TAGATTTCATTTATTACCTCCTTAC	P*vanZ* amplification with fluorescein
oMC1737	AGtGaattcgagctcggtacccggggatccCAGAAGAACAGTTTAAACTTTTAAAAG	P*clnR* Gibson assembly
oMC1738	CATTCCATGTGATGGTGATGGTGATGCATggcgccTTCATGCCTCCTTATTATATTAttg	P*clnR* Gibson assembly
oMC1739	CTCCAAAATACTACATAAATAA	P*mtlA* amplifciation
oMC1740	TATATCGATATGATTCCCTTTTG	P*mtlA* amplification
oMC1741	CAAATTAAAATAAAGCAATTTATA	P*CD1606* amplification
oMC1742	CTAGTGTATTAATACGATAGTAC	P*CD1606* amplification
oMC1743	TTGTTTAAGTATTAATTATGAGT	P*csfU* amplification
oMC1744	CGTCATTATATATAACGATTTATAC	P*csfU* amplification
oMC1745	ATGTTTCATCCCCTTTTTTAATC	P*cdd4/CD0668* amplification
oMC1746	CACCCTCCTTTAGTATACC	P*cdd4/CD0668* amplification
oMC1717	TTGAGTACTATAGGTGACCCAATGA	*mtlA (CD2334)* qPCR
oMC1718	CCTCTTTGTCCTGCTATTGCTTTA	*mtlA (CD2334)* qPCR
oMC1719	GGGTAAATGGTGGTATGGTACTTATT	*iorA (CD2381)* qPCR
oMC1720	AGCTTCTTGACTAGTTGATGGTTC	*iorA (CD2381)* qPCR
oMC1754	GGAATAATAGTTATGACTCATGGGAGTT	*CD0284* qPCR
oMC1755	AGTTTAATTGCTGCTGTTCTTTCTG	*CD0284* qPCR
tcdRqF	AGCAAGAAATAACTCAGTAGATGATT	*tcdR (CD0659*) qPCR[14]
tcdRqR	TTATTAAATCTGTTTCTCCCTCTTCA	*tcdR (CD0659*) qPCR[14]

[a] All sequences are listed 5’ to 3’. Underlined sequences denote restriction sites or intron retarget sites.
[b] Abbreviations: qPCR, quantitative PCR

Table S11. Plasmid construct details.

pMC602: The group II intron of pCE240 was targeted to *CD1617* at nucleotide 127 by splicing PCR using primers oMC1310, oMC1311, oMC1312, and EBSu as outlined in the TargeTron users manual (Sigma-Aldrich). The primers for intron retargeting were obtained by using the jpintronator algorithm. The group II *CD1617*-targeted intron was subcloned using the *BsrG*I and *Hind*III sites into pCE240.

pMC616: The 5.45 kb *Sph*I/*Sfo*I fragment from pMC602 was cloned as *Sph*I/*SnaB*I into pMC123.

pMC643: The group II intron of pCE240 was targeted to *CD1618* at nucleotide 217 by splicing PCR using primers oMC1319, oMC1320, oMC1321, and EBSu as outlined in the TargeTron users manual (Sigma-Aldrich). The primers for intron retargeting were obtained by using the jpintronator algorithm. The group II *CD1618*-targeted intron was subcloned using the *BsrG*I and *Hind*III sites into pCE240.

pMC645: The 5.45 kb *Sph*I/*Sfo*I fragment from pMC645 was cloned as *Sph*I/*SnaB*I into pMC123.

pMC649: The coding sequence of *CD1617-1619* and 300 bp upstream of *CD1617* was amplified using oMC1416 and oMC1476 and cloned into pSMB47 as *Bam*HI/*Sph*I.

pMC687: The coding sequence of *CD1234* was amplified with primers oMC1609 and oMC1610 and cloned into pMC211 as *BamH*I/*Pst*I.

pMC723: The *clnRAB* operon was amplified with an N-terminal 6x His tag using primers oMC1689 and oMC1476 and cloned as *BamH*I/*Sph*I into pMC123. Subsequently, P*clnR* (amplified using primers oMC1737 and oMC1738) was cloned by Gibson assembly as *BamH*I/*Sfo*I.

Table S12. Expression of ClnR-dependent genes in the *clnR* mutant complemented with HIS-ClnR.

Gene	BHIS			+ LL-37 (0.5 µg/ml)		
	630Δ*erm* pMC123	*clnR* pMC123	*clnR* pMC123 P*clnR::HIS-clnRAB*	630Δ*erm* pMC123	*clnR* pMC123	*clnR* pMC123 P*clnR::HIS-clnRAB*
clnA (CD630_16180)	1.0 ± 0.0[a]	37.4 ± 6.6	69.4 ± 20.3	81.6 ± 20.5	44.9 ± 8.0	236.5 ± 22.7
vanZ1 (CD630_12400)	1.0 ± 0.0	17.5 ± 2.4	4.1 ± 1.3	15.4. ± 2.8	21.8 ± 1.8	18.6 ± 2.3

[a]Relative expression levels were determined by qRT-PCR and are normalized to 630Δ*erm* + pMC123 in BHIS as described in methods. Values shown are the mean of 3 biological replicates ± standard error of the mean.

Additional References

1. Dalgaard P, Koutsoumanis K. Comparison of maximum specific growth rates and lag times estimated from absorbance and viable count data by different mathematical models. J Microbiol Methods 2001;43:183-96, NIHMS
2. Pope CF, McHugh TD, Gillespie SH. Methods to determine fitness in bacteria. Methods Mol Biol 2010;642:113-21, NIHMS
3. Wust J, Hardegger U. Transferable resistance to clindamycin, erythromycin, and tetracycline in *Clostridium difficile*. Antimicrob Agents Chemother 1983;23:784-6.PMCID: PMC184818
4. Hussain HA, Roberts AP, Mullany P. Generation of an erythromycin-sensitive derivative of Clostridium difficile strain 630 (630Deltaerm) and demonstration that the conjugative transposon Tn916DeltaE enters the genome of this strain at multiple sites. J Med Microbiol 2005;54:137-41, NIHMS
5. Stabler RA, He M, Dawson L, Martin M, Valiente E, Corton C, Lawley TD, Sebaihia M, Quail MA, Rose G, Gerding DN, Gibert M, Popoff MR, Parkhill J, Dougan G, Wren BW. Comparative genome and phenotypic analysis of *Clostridium difficile* 027 strains provides insight into the evolution of a hypervirulent bacterium. Genome Biology 2009;10:R102.PMCID: 2768977
6. Thomas CM, Smith CA. Incompatibility group P plasmids: genetics, evolution, and use in genetic manipulation. Annu Rev Microbiol 1987;41:77-101, NIHMS
7. Yanisch-Perron C, Vieira J, Messing J. Improved M13 phage cloning vectors and host strains: nucleotide sequences of the M13mp18 and pUC19 vectors. Gene 1985;33:103-19, NIHMS
8. Ho TD, Ellermeier CD. PrsW is required for colonization, resistance to antimicrobial peptides, and expression of extracytoplasmic function sigma factors in *Clostridium difficile*. Infect Immun 2011;79:3229-38.PMCID: 3147581
9. Manganelli R, Provvedi R, Berneri C, Oggioni MR, Pozzi G. Insertion vectors for construction of recombinant conjugative transposons in *Bacillus subtilis* and *Enterococcus faecalis*. FEMS Microbiol Lett 1998;168:259-68, NIHMS
10. McBride SM, Sonenshein AL. Identification of a genetic locus responsible for antimicrobial peptide resistance in *Clostridium difficile*. Infection and Immunity 2011;79:167-76.PMCID: 3019887
11. Edwards AN, Nawrocki KL, McBride SM. Conserved oligopeptide permeases modulate sporulation initiation in *Clostridium difficile*. Infect Immun 2014;82:4276-91.PMCID: 4187847
12. Dineen SS, McBride SM, Sonenshein AL. Integration of metabolism and virulence by *Clostridium difficile* CodY. J Bacteriol 2010;192:5350-62.PMCID: 2950512
13. Woods EC, Nawrocki KL, Suárez JM, McBride SM. The *Clostridium difficile* Dlt pathway is controlled by the ECF sigma factor, σV, in response to lysozyme. Infection and Immunity 2016;10.1128/iai.00207-16:IAI.00207-16, NIHMS
14. McKee RW, Mangalea MR, Purcell EB, Borchardt EK, Tamayo R. The second messenger cyclic Di-GMP regulates *Clostridium difficile* toxin production by controlling expression of *sigD*. J Bacteriol 2013;195:5174-85.PMCID: 3811590
