# Supplementary material for: The C. difficile clnRAB operon initiates adaptations to the host environment in response to LL-37
Source: PLoS Pathog. 2018 Aug 20;14(8):e1007153. doi: 10.1371/journal.ppat.1007153 (PMC6117091; doi:10.1371/journal.ppat.1007153)
Supplement: S6 Table — (PDF) [file ppat.1007153.s017.pdf]

Table S6. Relative expression of selected RNA-seq transcripts in *clnR* and *clnAB* mutants

Gene	BHIS					+ LL-37 (2 µg/ml)				
	630Δ*erm*	*clnR*	*clnAB*	*clnR* Tn::*clnRAB*	*clnAB* Tn::*clnRAB*	630Δ*erm*	*clnR*	*clnAB*	*clnR* Tn::*clnRAB*	*clnAB* Tn::*clnRAB*
cdd4 (CD630_06670)	1.0 ± 0.0[a]	0.9 ± 0.1	1.1 ± 0.2	0.9 ± 0.2	1.0 ± 0.2	7.1*± 2.4	**915.0***± 30.5	**1672.6*** ± 357	57.2*± 16.1	314.8*± 98.0
vanZ1 (CD630_12400)	1.0 ± 0.0	**25.2** ± 5.5	0.8 ± 0.1	0.8 ± 0.1	0.6 ± 0.0	36.9*± 6.0	**13.4** ± 5.8	**0.9** ± 0.2	**81.5*** ± 7.5	**0.6** ± 0.1
cstA (CD630_26000)	1.0 ± 0.0	0.9 ± 0.2	1.2 ± 0.1	0.6 ± 0.1	0.8 ± 0.2	6.7*± 1.5	2.3 ± 0.5	2.9 ± 1.2	36.3 ± 21.3	1.3 ± 0.2
sigU (csfU; CD630_18870)	1.0 ± 0.0	0.5 ± 0.2	1.3 ± 0.3	0.6 ± 0.1	0.7 ± 0.1	10.5 ± 6.8	2.0 ± 0.6	3.1 ± 1.4	**25.6***± 1.9	0.8 ± 0.1
sigT (csfT; CD630_06770)	1.0 ± 0.0	0.9 ± 0.1	1.0 ± 0.2	1.1 ± 0.1	1.1 ± 0.1	3.6*± 0.1	2.3 ± 1.1	1.2 ± 0.1	**25.3***± 1.7	1.2 ± 0.2
grdA (CD630_23520)	1.0 ± 0.0	0.9 ± 0.1	1.0 ± 0.0	0.9 ± 0.1	1.3 ± 0.2	16.5 ± 10.5	1.4 ± 0.1	1.9* ± 0.1	**1649.2** ± 814.6	1.6 ± 0.2
mtlA (CD630_23340)	1.0 ± 0.0	0.9 ± 0.4	3.3 ± 2.1	0.6 ± 0.2	0.5 ± 0.1	1.0 ± 0.3	1.2 ± 0.7	5.5 ± 4.3	0.6 ± 0.2	0.7 ± 0.4
iorA (CD630_23810)	1.0 ± 0.0	**0.5** ± 0.1	0.7 ± 0.1	0.7 ± 0.0	1.1 ± 0.2	16.8 ± 8.4	2.1*± 0.4	20.5 ± 7.3	**112.4***± 19.9	2.8*± 0.3
CD630_02840	1.0 ± 0.0	1.2 ± 0.1	1.1 ± 0.1	1.1 ± 0.2	1.3 ± 0.2	29.3 ± 18.8	2.2 ± 0.5	2.1* ± 0.2	602.6 ± 455.6	2.8*± 0.1

[a]Relative expression levels were determined by qRT-PCR and are normalized to 630Δ*erm* in BHIS as described in methods. Values shown are the mean of at least 3 biological replicates ± standard error of the mean. Data were analyzed by one-way ANOVA and Dunnett' s multiple comparisons test for comparisons to 630Δ*erm* in the same condition or by Student's *t* test with Holm-Sidak correction for comparisons of the same strain with/without LL-37. Bolded values indicate an adjusted p value < 0.05 comparing to 630Δ*erm* in the same condition; * indicates an adjusted p value < 0.05 comparing the same strain with/without LL-37.
