# Supplementary material for: The C. difficile clnRAB operon initiates adaptations to the host environment in response to LL-37
Source: PLoS Pathog. 2018 Aug 20;14(8):e1007153. doi: 10.1371/journal.ppat.1007153 (PMC6117091; doi:10.1371/journal.ppat.1007153)
Supplement: S7 Table — (PDF) [file ppat.1007153.s018.pdf]

Table S7. Maximum doubling times for 630Δ*erm*, *clnR, clnAB, clnR* Tn::*clnRAB,* and *clnAB* Tn::*clnRAB* in minimal media supplemented with metabolites, with or without LL-37

MM+[a]	strain	No LL-37 Doubling time (min)[b]	LL-37[c] Doubling time (min)[b]
--	630Δ*erm*	73.2 ± 3.4	82.2 ± 5.1
	clnR	76.3 ± 2.6	74.1 ± 4.8
	clnAB	73.5 ± 2.3	77.1 ± 5.3
	clnR Tn::*clnRAB*	71.6 ± 2.5	§84.8 ± 4.5
	clnAB Tn::*clnRAB*	71.7 ± 2.0	76.4 ± 2.6
Glucose	630Δ*erm*	66.2 ± 3.7	67.7 ± 4.9
	clnR	68.4 ± 3.5	74.0 ± 1.7
	clnAB	*61.4 ± 3.3	62.1 ± 2.2
	clnR Tn::*clnRAB*	64.6 ± 3.7	76.4 ± 5.4
	clnAB Tn::*clnRAB*	63.6 ± 2.5	70.8 ± 4.8
Fructose	630Δ*erm*	64.8 ± 4.1	70.2 ± 3.9
	clnR	70.6 ± 1.7	72.4 ± 0.9
	clnAB	63.9 ± 3.5	65.1 ± 2.0
	clnR Tn::*clnRAB*	67.8 ± 2.5	80.4 ± 7.4
	clnAB Tn::*clnRAB*	67.2 ± 2.6	69.8 ± 2.5
Mannose	630Δ*erm*	71.0 ± 5.2	70.3 ± 5.5
	clnR	70.5 ± 2.9	78.4 ± 4.9
	clnAB	65.6 ± 3.7	66.8 ± 4.5
	clnR Tn::*clnRAB*	72.4 ± 4.6	80.4 ± 8.3
	clnAB Tn::*clnRAB*	68.9 ± 3.4	72.6 ± 3.1
Mannitol	630Δ*erm*	60.0 ± 2.1	69.9 ± 3.4
	clnR	63.7 ± 0.4	67.3 ± 1.5
	clnAB	60.1 ± 3.2	58.3 ± 5.5
	clnR Tn::*clnRAB*	64.5 ± 2.6	75.6 ± 5.3
	clnAB Tn::*clnRAB*	*62.4 ± 0.7	66.4 ± 1.8
NAG	630Δ*erm*	66.6 ± 2.7	§91.5 ± 5.7
	clnR	81.8 ± 1.5	86.6 ± 2.5
	clnAB	70.9 ± 0.8	§80.3 ± 1.0
	clnR Tn::*clnRAB*	73.6 ± 3.1	111.1 ± 16.5
	clnAB Tn::*clnRAB*	*72.4 ± 1.4	§83.6 ± 1.7
EA	630Δ*erm*	78.4 ± 4.0	90.7 ± 7.9
	clnR	*89.9 ± 3.2	84.9 ± 4.6
	clnAB	87.7 ± 7.0	79.6 ± 9.0
	clnR Tn::*clnRAB*	86.2 ± 4.7	93.4 ± 12.2
	clnAB Tn::*clnRAB*	85.9 ± 7.2	84.0 ± 6.7

[a]Strains were grown in MM with additional nutrients as listed: 30mM glucose, 30 mM fructose, 30 mM mannose, 30 mM N-acetylglucosamine (NAG), 30 mM mannitol, 30 mM ethanolamine (EA)
[b]Doubling time was calculated during the 2 hour period of maximal growth after the conclusion of peptide-fueled growth, where $t_d = \ln(2)/\mu$, where $\mu = (\ln(OD_{t2}) - \ln(OD_{t1}))/(t_2 - t_1)$[1, 2]. Values shown are the average of at least three independent experiments ± standard deviation.
[c]LL-37, 0.5 µg/ml
§To compare +/- LL-37 for each strain/substrate (across rows), data were analyzed by Student's *t* test (**§**indicates $p < 0.05$).
To compare the growth of strains in each condition (within boxes), data were analyzed by one-way ANOVA with Dunnett's test for multiple comparisons (indicates adjusted $p < 0.05$)
