# Supplementary material for: The C. difficile clnRAB operon initiates adaptations to the host environment in response to LL-37
Source: PLoS Pathog. 2018 Aug 20;14(8):e1007153. doi: 10.1371/journal.ppat.1007153 (PMC6117091; doi:10.1371/journal.ppat.1007153)
Supplement: S8 Table — (PDF) [file ppat.1007153.s019.pdf]

Table S8. Expression of toxin regulation-associated genes

**Gene**	**Fold-change in expression LL-37**[a, b]
sigD	0.95 ± 0.08
tcdR	1.47 ± 0.44
ilvC[c]	**2.21** ± 0.20
CD0341[d]	2.54 ± 0.59

[a]Fold-change determined by qRT-PCR and normalized to 630Δ*erm* grown in BHIS alone. Concentration of LL-37 = 2 μg/ml. Values are the mean of three replicates ± standard error of the mean.
[b]Bolded values indicate significant difference (P value < 0.05) from 630Δ*erm* grown in BHIS without LL-37 and analyzed by Student's two-tailed *t*-test.
[c]*ilvC* indicates CodY activity.
[d]*CD0341* indicates CcpA activity.
