# Supplementary material for: The C. difficile clnRAB operon initiates adaptations to the host environment in response to LL-37
Source: PLoS Pathog. 2018 Aug 20;14(8):e1007153. doi: 10.1371/journal.ppat.1007153 (PMC6117091; doi:10.1371/journal.ppat.1007153)
Supplement: S9 Table — (PDF) [file ppat.1007153.s020.pdf]

Table S9. Plasmids and Strains

Plasmid or Strain		Relevant genotype or features	Source, construction or reference
**Strains**			
E. coli			
	HB101	F$^-$ *mcrB mrr hsdS20*(r_B^- m_B^-) *recA13 leuB6 ara-14 proA2 lacY1 galK2 xyl-5 mtl-1 rpsL20*	B. Dupuy
	MC101	HB101 pRK24	B. Dupuy
	MC135	HB101 pRK24 pMC123	(McBride and Sonenshein, 2011)
	MC881	HB101 pRK24 pMC616	
	MC932	HB101 pRK24 pMC645	
	MC1122	HB101 pRK24 pMC723	
B. subtilis	BS49	*Tn*916	
	MC951	BS49 *Tn*916::*CD1617-1619*	
C. difficile			
	630	Clinical isolate	[3]
	630Δ*erm*	Erm^S derivative of strain 630	N. Minton [4]
	R20291	Clinical isolate	[5]
	MC324	630Δ*erm* pMC123	(Edwards et al., 2014)
	MC885	630Δ*erm CD1617::ermB*	
	MC935	630Δ*erm CD1618::ermB*	
	MC950	MC885 *Tn*916::*CD1617-1619*	
	MC953	MC935 *Tn*916::*CD1617-1619*	
	MC1123	MC885 pMC123	
	MC1131	MC885 pMC723	
**Plasmids**	pRK24	Tra$^+$, Mob$^+$; *bla, tet*	[6]
	pCR2.1	*bla, kan*	Invitrogen
	pUC19	Cloning vector; *bla*	[7]
	pCE240	*C. difficile* TargeTron→ construct based on pJIR750ai (group II intron, *ermB::*RAM, *ltrA*); *catP*	C. Ellermeier; [8]
	pSMB47	Tn*916* integrational vector; CmR, ErmR	[9]
	pMC123	*E. coli-C. difficile* shuttle vector; *bla, catP*	
	pMC577	pCR2.1 with *clnR*-targeted intron	
	pMC602	pCE240 with *clnR*-targeted intron	
	pMC616	pMC123 with *clnR*-targeted intron (~nt 127, *ermB::*RAM *ltrA catP*	
	pMC643	pCE240 with *clnA*-targeted intron	
	pMC645	pMC123 with *clnA*-targeted intron (~nt 217, *ermB*::RAM *ltrA catP*	
	pMC649	pSMB47 Tn*916*::*CD1617-1619*	
	pMC723	pMC123 with P_{clnR}::His-*clnRAB*	
