# Supplementary material for: The C. difficile clnRAB operon initiates adaptations to the host environment in response to LL-37
Source: PLoS Pathog. 2018 Aug 20;14(8):e1007153. doi: 10.1371/journal.ppat.1007153 (PMC6117091; doi:10.1371/journal.ppat.1007153)
Supplement: S10 Table — (PDF) [file ppat.1007153.s021.pdf]

Table S10. Oligonucleotides

Primer	Sequence[a]	Purpose, source, or reference[b]
oMC44	CTAGCTGCTCCTATGTCTCACATC	*rpoC* (*CD0067*) qPCR [10]
oMC45	CCAGTCTCTCCTGGATCAACTA	*rpoC* (*CD0067*) qPCR [10]
oMC112	GGCAAATGTAAGATTTCGTACTCA	*tcdB* (*CD0660*) qPCR [11]
oMC113	TCGACTACAGTATTCTCTGAC	*tcdB* (*CD0660*) qPCR [11]
oMC152	GTTATGGAAGTCAAGGACATGCAC	*ilvC* (*CD1565*) qPCR [12]
oMC153	GCTTCTGCTACACTCTTAACTTCA	*ilvC* (*CD1565*) qPCR [12]
oMC178	CTTGAGTTAAATCTTGTGCAGTCA	*csfU* (*CD1887*) qPCR
oMC179	GGTGATAATAGTGAATGATGCTCGG	*csfU* (*CD1887*) qPCR
oMC189	TGCCTCTTGTAAAGAGTATAGCA	*sigD* (*CD0266*) qPCR [11]
oMC190	GCATCAATCAATCCAATGACTCCAC	*sigD* (*CD0266*) qPCR [11]
oMC242	TCCACAAGGAGCTGTATATGGT	*cdd4* (*CD0667*) qPCR
oMC243	GTGGGTTTAGCAAGTCCAAGAA	*cdd4* (*CD0667*) qPCR
oMC547	TGGATAGGTGGAGAAGTCAGT	*tcdA* (*CD0663*) qPCR [11]
oMC548	GCTGTAATGCTTCAGTGGTAGA	*tcdA* (*CD0663*) qPCR [11]
oMC683	GTATCTGACAACATCAATTGCCTAAA	*CD0341* qPCR [11]
oMC684	TCAGCTTGAGATTCAATTTCTTCATT	*CD0341* qPCR [11]
oMC815	TGGATTCTCTTAAGGAAGAACAATACTTTA	*sigT* (*CD0677*) qPCR [13]
oMC816	CCTTAACTTCATCTACTGAATAACCTTCA	*sigT* (*CD0677*) qPCR [13]
oMC1249	GTCGAGGATCCGATGACAAGTTATTGGAATACACAG	P*spo0A* amplification
oMC1290	GAATGGGAACTTGATAATAACAAACC	check *CD1617-1618* co-transcription ; *clnR* (*CD1617*) qPCR
oMC1291	AAGTTCTGTTAGAGCCTTTTGC	check *CD1616-1617* co-transcription; *clnR* (*CD1617*) qPCR
oMC1292	AGGTGTAAACAAGAGTTATGGAAC	check *CD1618-1619* co-transcription ; *clnA* (*CD1618*) qPCR
oMC1293	TCTATGGATGGTTTCATTCCATTTATC	check *CD1617-1618* co-transcription ; *clnA* (*CD1618*) qPCR
oMC1294	AAGCAAGTGGAAGAATATTTATACCG	*clnB* (*CD1619*) qPCR
oMC1295	ACATTAAATAACCTTCATCCCCC	*clnB* (*CD1619*) qPCR
oMC1297	CACTGCAGTTTTATCCATTTTATAATTC	screening for Targetron insertion in *clnR*
oMC1310	AAAAGCTTTTGCAACCCACGTCGATCGTGAACCGCATCTTCTGGTGCGCCCAGATAGGGT	*clnR (CD1617)* intron retargeting
oMC1311	CAGATTGTACAAATGTGGTGATAACAGATAAGTCCTTCTGCTTAACTTACCTTTCTTTGT	*clnR (CD1617)* intron retargeting
oMC1312	CGCAAGTTTCTAATTTCGGTTTGCGGTCGATAGAGGAAAGTGTCT	*clnR (CD1617)* intron retargeting
oMC1319	AAAAGCTTTTGCAACCCACGTCGATCGTGAAAAAATAGTTTCAGTGCGCCCAGATAGGGT	*clnA (CD1618)* intron retargeting
oMC1320	CAGATTGTACAAATGTGGTGATAACAGATAAGTCGTTTCATATAACTTACCTTTCTTTGT	*clnA (CD1618)* intron retargeting
oMC1321	CGCAAGTTTCTAATTTCGGTTATTTTTCGATAGAGGAAAGTGTCT	*clnA (CD1618)* intron retargeting
oMC1383	GTAGAAGGAGCAGAGGTTGTTT	*grdA (CD2352)* qPCR
oMC1384	TCAGCAGCATCTTTAACTCTGT	*grdA (CD2352)* qPCR
oMC1393	TGAAACCATGAATCTTAGAAGCATAAAC	*vanZ (CD1240)* qPCR
oMC1394	CACATATATCCCAAATGGTACAAATATAGC	*vanZ (CD1240)* qPCR
oMC1410	GTGGGATCCGCTAAAACTTATTACAG	*clnA* (*CD1618*) cloning
oMC1416	GTGGGATCCAGAAGAACAGTTTAA	P*clnR* cloning
oMC1427	GTTTGGAAAGCCAATGCCAA	check *CD1616-1617* co-transcription
oMC1467	TAGCAGAAGATGCGGAAGTTAAT	*clnR* (*CD1617*) qPCR
oMC1473	GTTACAAATCTTCCTTTAGTTCTCTGAC	*clnR* (*CD1617*) qPCR
oMC1476	GCGCATGCATTACTCAAAAGATAGCT	*clnRAB* cloning
oMC1483	CTGGGTCAACACCACCTATAG	verify *clnA (CD1618)* disruption
oMC1493	GTTAGAAGAGCAAATGAGATGATTAAAAGC	*clnA* (*CD1618*) qPCR
oMC1614	GTGTACTCCACCAGCAAAGA	*cstA* (*CD2600*) qPCR
oMC1615	GCAGGGTTAGGTCCGATATTT	*cstA* (*CD2600*) qPCR
oMC1684	GCGGAATTCCTAAAAAGTAATTGACATATACTTTG	P*clnR* cloning
oMC1689	GTGGGATCCGGCGCCATGCATCACCATCACCATCACATGGAATGGGAACTTGATAATAAC	*clnR* cloning with His-tag

oMC1690	GTGGGCGCCGTTCATGCCTCCTTATTA	P*clnR* cloning
oMC1691	FAM-CTAAAAAGTAATTGACATATACTTTG	P*clnR* amplification with fluorescein
oMC1692	FAM-CATGCCTCCTTATTATATTATTG	P*clnR* amplification with fluorescein
oMC1700	FAM-AGTTTGTGCAGTTTCTGAA	P*iorA* amplification with fluorescein
oMC1701	FAM-ACTACAATTATTAAATTCATAGATG	P*iorA* amplification with fluorescein
oMC1702	FAM-CTCCAAAATACTACATAAATAA	P*mtlA* amplification with fluorescein
oMC1703	FAM-TATATCGATATGATTCCCTTTTG	P*mtlA* amplification with fluorescein
oMC1704	FAM-CAAATTAAAATAAAGCAATTTATA	P*CD1606* amplification with fluorescein
oMC1705	FAM-CTAGTGTATTAATACGATAGTAC	P*CD1606* amplification with fluorescein
oMC1706	FAM-TTGTTTAAGTATTAATTATGAGT	P*csfU* amplification with fluorescein
oMC1707	FAM-CGTCATTATATATAACGATTTATAC	P*csfU* amplification with fluorescein
oMC1710	FAM-ATGTTTCATCCCCTTTTTTAATC	P*cdd4/CD0668* amplification with fluorescein
oMC1711	FAM-CACCCTCCTTTAGTATACC	P*cdd4/CD0668* amplification with fluorescein
oMC1712	FAM-CAATATTAATTTATTTTTAAAAAATAG	P*tcdA* amplification with fluorescein
oMC1713	FAM-AGTATTATTATTTTTGATAATAAATC	P*tcdA* amplification with fluorescein
oMC1714	FAM-CTTGTAATAAAATAAAGATTTAAGTG	P*grdE* amplification with fluorescein
oMC1715	FAM-CACCTCCTGTTATTTAATTTG	P*grdE* amplification with fluorescein
oMC1716	CACCAATAATTTTATTATTTTGTATTATTG	P*spo0A* amplification
oMC1735	AGAAAGATATGAAATACTACAATAGC	P*vanZ* amplification with fluorescein
oMC1736	TAGATTTCATTTATTACCTCCTTAC	P*vanZ* amplification with fluorescein
oMC1737	AGtGaattcgagctcggtacccggggatccCAGAAGAACAGTTTAAACTTTTAAAAG	P*clnR* Gibson assembly
oMC1738	CATTCCATGTGATGGTGATGGTGATGCATggcgccTTCATGCCTCCTTATTATATTAttg	P*clnR* Gibson assembly
oMC1739	CTCCAAAATACTACATAAATAA	P*mtlA* amplifciation
oMC1740	TATATCGATATGATTCCCTTTTG	P*mtlA* amplification
oMC1741	CAAATTAAAATAAAGCAATTTATA	P*CD1606* amplification
oMC1742	CTAGTGTATTAATACGATAGTAC	P*CD1606* amplification
oMC1743	TTGTTTAAGTATTAATTATGAGT	P*csfU* amplification
oMC1744	CGTCATTATATATAACGATTTATAC	P*csfU* amplification
oMC1745	ATGTTTCATCCCCTTTTTTAATC	P*cdd4/CD0668* amplification
oMC1746	CACCCTCCTTTAGTATACC	P*cdd4/CD0668* amplification
oMC1717	TTGAGTACTATAGGTGACCCAATGA	*mtlA (CD2334)* qPCR
oMC1718	CCTCTTTGTCCTGCTATTGCTTTA	*mtlA (CD2334)* qPCR
oMC1719	GGGTAAATGGTGGTATGGTACTTATT	*iorA (CD2381)* qPCR
oMC1720	AGCTTCTTGACTAGTTGATGGTTC	*iorA (CD2381)* qPCR
oMC1754	GGAATAATAGTTATGACTCATGGGAGTT	*CD0284* qPCR
oMC1755	AGTTTAATTGCTGCTGTTCTTTCTG	*CD0284* qPCR
tcdRqF	AGCAAGAAATAACTCAGTAGATGATT	*tcdR (CD0659*) qPCR[14]
tcdRqR	TTATTAAATCTGTTTCTCCCTCTTCA	*tcdR (CD0659*) qPCR[14]

[a] All sequences are listed 5’ to 3’. Underlined sequences denote restriction sites or intron retarget sites.
[b] Abbreviations: qPCR, quantitative PCR
