# Supplementary material for: The C. difficile clnRAB operon initiates adaptations to the host environment in response to LL-37
Source: PLoS Pathog. 2018 Aug 20;14(8):e1007153. doi: 10.1371/journal.ppat.1007153 (PMC6117091; doi:10.1371/journal.ppat.1007153)
Supplement: S11 Table — (PDF) [file ppat.1007153.s022.pdf]

Table S11. Plasmid construct details.

pMC602: The group II intron of pCE240 was targeted to *CD1617* at nucleotide 127 by splicing PCR using primers oMC1310, oMC1311, oMC1312, and EBSu as outlined in the TargeTron users manual (Sigma-Aldrich). The primers for intron retargeting were obtained by using the jpintronator algorithm. The group II *CD1617*-targeted intron was subcloned using the *BsrG*I and *Hind*III sites into pCE240.

pMC616: The 5.45 kb *Sph*I/*Sfo*I fragment from pMC602 was cloned as *Sph*I/*SnaB*I into pMC123.

pMC643: The group II intron of pCE240 was targeted to *CD1618* at nucleotide 217 by splicing PCR using primers oMC1319, oMC1320, oMC1321, and EBSu as outlined in the TargeTron users manual (Sigma-Aldrich). The primers for intron retargeting were obtained by using the jpintronator algorithm. The group II *CD1618*-targeted intron was subcloned using the *BsrG*I and *Hind*III sites into pCE240.

pMC645: The 5.45 kb *Sph*I/*Sfo*I fragment from pMC645 was cloned as *Sph*I/*SnaB*I into pMC123.

pMC649: The coding sequence of *CD1617-1619* and 300 bp upstream of *CD1617* was amplified using oMC1416 and oMC1476 and cloned into pSMB47 as *Bam*HI/*Sph*I.

pMC687: The coding sequence of *CD1234* was amplified with primers oMC1609 and oMC1610 and cloned into pMC211 as *BamH*I/*Pst*I.

pMC723: The *clnRAB* operon was amplified with an N-terminal 6x His tag using primers oMC1689 and oMC1476 and cloned as *BamH*I/*Sph*I into pMC123. Subsequently, P*clnR* (amplified using primers oMC1737 and oMC1738) was cloned by Gibson assembly as *BamH*I/*Sfo*I.
