# Supplementary material for: The C. difficile clnRAB operon initiates adaptations to the host environment in response to LL-37
Source: PLoS Pathog. 2018 Aug 20;14(8):e1007153. doi: 10.1371/journal.ppat.1007153 (PMC6117091; doi:10.1371/journal.ppat.1007153)
Supplement: S12 Table — (PDF) [file ppat.1007153.s023.pdf]

Table S12. Expression of ClnR-dependent genes in the *clnR* mutant complemented with HIS-ClnR.

Gene	BHIS			+ LL-37 (0.5 µg/ml)		
	630Δ*erm* pMC123	*clnR* pMC123	*clnR* pMC123 P*clnR::HIS-clnRAB*	630Δ*erm* pMC123	*clnR* pMC123	*clnR* pMC123 P*clnR::HIS-clnRAB*
clnA (CD630_16180)	1.0 ± 0.0[a]	37.4 ± 6.6	69.4 ± 20.3	81.6 ± 20.5	44.9 ± 8.0	236.5 ± 22.7
vanZ1 (CD630_12400)	1.0 ± 0.0	17.5 ± 2.4	4.1 ± 1.3	15.4. ± 2.8	21.8 ± 1.8	18.6 ± 2.3

[a]Relative expression levels were determined by qRT-PCR and are normalized to 630Δ*erm* + pMC123 in BHIS as described in methods. Values shown are the mean of 3 biological replicates ± standard error of the mean.

Additional References

1. Dalgaard P, Koutsoumanis K. Comparison of maximum specific growth rates and lag times estimated from absorbance and viable count data by different mathematical models. J Microbiol Methods 2001;43:183-96, NIHMS
2. Pope CF, McHugh TD, Gillespie SH. Methods to determine fitness in bacteria. Methods Mol Biol 2010;642:113-21, NIHMS
3. Wust J, Hardegger U. Transferable resistance to clindamycin, erythromycin, and tetracycline in *Clostridium difficile*. Antimicrob Agents Chemother 1983;23:784-6.PMCID: PMC184818
4. Hussain HA, Roberts AP, Mullany P. Generation of an erythromycin-sensitive derivative of Clostridium difficile strain 630 (630Deltaerm) and demonstration that the conjugative transposon Tn916DeltaE enters the genome of this strain at multiple sites. J Med Microbiol 2005;54:137-41, NIHMS
5. Stabler RA, He M, Dawson L, Martin M, Valiente E, Corton C, Lawley TD, Sebaihia M, Quail MA, Rose G, Gerding DN, Gibert M, Popoff MR, Parkhill J, Dougan G, Wren BW. Comparative genome and phenotypic analysis of *Clostridium difficile* 027 strains provides insight into the evolution of a hypervirulent bacterium. Genome Biology 2009;10:R102.PMCID: 2768977
6. Thomas CM, Smith CA. Incompatibility group P plasmids: genetics, evolution, and use in genetic manipulation. Annu Rev Microbiol 1987;41:77-101, NIHMS
7. Yanisch-Perron C, Vieira J, Messing J. Improved M13 phage cloning vectors and host strains: nucleotide sequences of the M13mp18 and pUC19 vectors. Gene 1985;33:103-19, NIHMS
8. Ho TD, Ellermeier CD. PrsW is required for colonization, resistance to antimicrobial peptides, and expression of extracytoplasmic function sigma factors in *Clostridium difficile*. Infect Immun 2011;79:3229-38.PMCID: 3147581
9. Manganelli R, Provvedi R, Berneri C, Oggioni MR, Pozzi G. Insertion vectors for construction of recombinant conjugative transposons in *Bacillus subtilis* and *Enterococcus faecalis*. FEMS Microbiol Lett 1998;168:259-68, NIHMS
10. McBride SM, Sonenshein AL. Identification of a genetic locus responsible for antimicrobial peptide resistance in *Clostridium difficile*. Infection and Immunity 2011;79:167-76.PMCID: 3019887
11. Edwards AN, Nawrocki KL, McBride SM. Conserved oligopeptide permeases modulate sporulation initiation in *Clostridium difficile*. Infect Immun 2014;82:4276-91.PMCID: 4187847
12. Dineen SS, McBride SM, Sonenshein AL. Integration of metabolism and virulence by *Clostridium difficile* CodY. J Bacteriol 2010;192:5350-62.PMCID: 2950512
13. Woods EC, Nawrocki KL, Suárez JM, McBride SM. The *Clostridium difficile* Dlt pathway is controlled by the ECF sigma factor, σV, in response to lysozyme. Infection and Immunity 2016;10.1128/iai.00207-16:IAI.00207-16, NIHMS
14. McKee RW, Mangalea MR, Purcell EB, Borchardt EK, Tamayo R. The second messenger cyclic Di-GMP regulates *Clostridium difficile* toxin production by controlling expression of *sigD*. J Bacteriol 2013;195:5174-85.PMCID: 3811590
